# Safety and effectiveness of acellular pertussis vaccination during pregnancy: a systematic review

**DOI:** 10.1186/s12879-020-4824-3

**Published:** 2020-02-13

**Authors:** Sabine Vygen-Bonnet, Wiebke Hellenbrand, Edeltraut Garbe, Rüdiger von Kries, Christian Bogdan, Ulrich Heininger, Marianne Röbl-Mathieu, Thomas Harder

**Affiliations:** 1 Immunization Unit, Robert Koch Institute, Seestrasse 10, 13353 Berlin, Germany; 2 Leibniz Institute for Prevention Research and Epidemiology – BIPS, Bremen, Germany; 3 Institute of Social Pediatrics and Adolescent Medicine, Division of Pediatric Epidemiology, Ludwig-Maximilians-University, Munich, Germany; 4 Institut für klinische Mikrobiologie, Immunologie und Hygiene, Universitätsklinikum Erlangen und Friedrich-Alexander-Universität (FAU) Erlangen-Nürnberg, Erlangen, Germany; 5 Medical Immunology Campus Erlangen, FAU Erlangen-Nürnberg, Erlangen, Germany; 6 University of Basel Children’s Hospital, Infectious Diseases and Vaccinology, Basel, Switzerland; 7 Working Group on Immunization of the federal state of Bavaria (LAGI), Munich, Germany; 8 Private practice for gynecology/obstetrics, Munich, Germany

**Keywords:** Tdap, Acellular pertussis vaccine, Pertussis, Pregnancy, Chorioamnionitis

## Abstract

**Background:**

Infants < 3 months of age are at highest risk for developing severe complications after pertussis. The majority of pregnant women has low concentrations of pertussis-specific antibodies and thus newborns are insufficiently protected by maternally transferred antibodies. Acellular pertussis vaccination during pregnancy was recently implemented in various countries. Here, we assessed the evidence for safety and effectiveness of pertussis vaccination during pregnancy.

**Methods:**

We searched Medline, Embase, and ClinicalTrials.gov from January 1st 2010 to January 10th 2019. We assessed risk of bias (ROB) using the Cochrane ROB tool and ROBINS-I. We evaluated the quality of evidence using the GRADE approach.

**Results:**

We identified 1273 articles and included 22 studies (14 for safety; 8 for effectiveness), comprising 1.4 million pregnant women in safety studies and 855,546 mother-infant-pairs in effectiveness studies. No significant differences between vaccinated and unvaccinated women and their infants were observed for safety outcomes with the exception of fever and chorioamnionitis. Compared to no vaccination, three studies showed a significantly increased relative risk for the presence of the ICD-9 code for chorioamnionitis in electronic patient data after pertussis vaccination. However, no study reported an increased risk for clinical sequelae of chorioamnionitis after vaccination during pregnancy, such as preterm birth or neonatal intensive care unit admission. Vaccine effectiveness against pertussis in infants of immunized mothers ranged from 69 to 91% for pertussis prevention, from 91 to 94% for prevention of hospitalization and was 95% for prevention of death due to pertussis. Risk of bias was serious to critical for safety outcomes and moderate to serious for effectiveness outcomes. GRADE evidence quality was moderate to very low, depending on outcome.

**Conclusion:**

Although an increased risk for a diagnosis of fever and chorioamnionitis was detected in pregnant women after pertussis vaccination, there was no association with a higher frequency of clinically relevant sequelae. Vaccine effectiveness for prevention of infant pertussis, hospitalization and death is high. Pertussis vaccination during pregnancy has an overall positive benefit-risk ratio. In view of the overall quality of available evidence ongoing surveillance of chorioamnionitis and its potential sequelae is recommended when pertussis vaccination in pregnancy is implemented.

**Trial registration:**

PROSPERO CRD42018087814, CRD42018090357.

## Background

Pertussis is a vaccine-preventable bacterial respiratory infection leading to high morbidity, especially in young infants. Disease burden of pertussis remains significant despite high vaccination coverage in children in western countries [1]. In Germany, annual pertussis incidence ranged from 11 to 20 per 100,000 inhabitants during the years 2013–2018 [2]. Young infants < 6 months of age are at increased risk of pertussis related complications, such as otitis media, pneumonia, apnea, encephalopathy, as well as pulmonary hypertension which is caused by extreme lymphocytosis [3]. Severe and potentially lethal complications are most common in infants < 2 months of age [4]. In Germany, mean annual incidence of pertussis among infants aged ≤3 months was 80 per 100,000 during the past 5 years, while hospitalization rate in those young infants was > 75% [RKI, surveillance data, unpublished]. A recent German capture-recapture study suggests that incidences based on statutory surveillance are substantially underestimated by 39% [5]. Since the introduction of nationwide mandatory pertussis reporting in Germany in 2013, two pertussis-related deaths were notified in infants 2 and 4 months of age [6] [RKI, surveillance data, unpublished]. Due to young age, this vulnerable group cannot benefit from direct effects of vaccination. Studies have shown that the majority of pregnant women in western countries have insufficient concentrations of pertussis-specific antibodies to confer protection to the newborn via diaplacentally transferred maternal antibodies [7–10]. In contrast, vaccination during pregnancy results in high levels of antibodies in the mother and the newborn [11, 12]. Therefore, vaccination of pregnant women with an acellular pertussis vaccine has been introduced in a number of countries, including the United Kingdom, USA, Belgium, Switzerland, Spain and Australia [13–19].

So far, six systematic reviews have investigated the effectiveness and/or safety of pertussis vaccination during pregnancy [4, 20–24]. Importantly, however, a number of new studies on this topic were published only recently, and not all of these reviews addressed the entire spectrum of clinically relevant outcomes comprising safety as well as effectiveness for mother and child. Furthermore, some of the earlier reviews did not use the most advanced methodological tools recommended to address risk of bias and evidence quality, both being of key importance for decision-making regarding the implementation of vaccine programs during pregnancy.

We therefore performed a systematic review assessing the evidence for safety and effectiveness of pertussis vaccination during pregnancy.

## Methods

### Search strategy and selection criteria

The protocols of this systematic review were published in the Prospective Register for Systematic Reviews (PROSPERO; registration no, CRD42018087814 (for safety), CRD42018090357 (for effectiveness)). The review was performed according to the guidelines in the Preferred Reporting Items for Systematic Reviews and Meta-analyses (PRISMA) statement [25].

To be eligible, a study had to match the following PICO (population, intervention, comparator, outcome) criteria:

P – pregnant women and their newborns.

I – vaccination with an acellular pertussis component-containing vaccine during pregnancy.

C – placebo or no vaccination or vaccination with other, not pertussis component-containing vaccines, e.g. tetanus, tetanus-diphtheria, or influenza vaccination (only for effectiveness outcomes).

O – *efficacy/effectiveness:* (1) laboratory-confirmed pertussis in infant ≤3 months of age; (2) hospitalization due to (1); (3) death due to (1);

O – *safety:* (4) fever (≥38 °C) in pregnant woman; (5) pre-eclampsia/eclampsia; (6) chorioamnionitis; (7) preterm birth; (8) stillbirth; (9) low birth weight; (10) malformation; (11) neonatal intensive care unit (NICU) admission; (12) neonatal sepsis; (13) neonatal death.

Electronic databases searched were MEDLINE and EMBASE (date of initial search: 26 February 2018; last update: 10 January 2019). For details on the complete search strategy, see Additional file 1: Figure S1. Additionally, the Cochrane Data Base of Clinical Trials was searched, and a search in ClinicalTrials.gov was conducted for unpublished or ongoing trials. Electronic searches were complemented by manually screening reference lists of all identified studies and those of identified reviews. Search results (titles, abstracts, full texts) were independently assessed by three investigators (WH, TH, SVB). Differences were discussed until a consensus was reached.

Search was limited to studies published from 01 January 2010 onwards. We did not make restrictions with regard to setting, language or publication status (published/unpublished).

### Data extraction

Three independent reviewers (WH, TH, SVB) used standardized forms to extract study characteristics from eligible studies and to assess risk of bias. In case of disagreement, a final decision was made by consensus. The following data were extracted: study location, setting, study design, study period, participants, intervention, comparator, study size, outcomes, study sponsorship, conflict of interests, number (proportion) of vaccinated participants with outcome, number (proportion) of control participants with outcome, unadjusted estimates, adjusted estimates, and confounders.

### Assessment of risk of bias and quality of evidence

For randomized controlled trials (RCTs), the Cochrane risk of bias tool was used to assess the following domains: random sequence generation, allocation concealment, blinding of participants and personnel, blinding of outcome assessment, incomplete outcome data, selective reporting, and other bias [26]. RCTs were categorized as being at “high risk”, “low risk” or “unclear risk” of bias. For non-randomized studies, the ROBINS-I tool was used, comprising the following domains: bias due to confounding, bias in selection of participants into the study, bias in classification of interventions, bias due to deviations from intended interventions, bias due to missing data, bias in measurement of outcomes, and bias in selection of the reported results [27]. Risk of bias was categorized as being “low risk”, “moderate risk”, “serious risk” or “critical risk”.

The methodology of the GRADE (Grading of Recommendations Assessment, Development and Evaluation) working group and the software “GRADE profiler” were used to assess the quality of evidence [28, 29].

### Statistical analysis

Abstracted data were aggregated in tables. Risk ratios, odds ratios, risk differences and corresponding 95% confidence intervals (95% CI) were either calculated or extracted from the publications. A *p*-value <.05 was considered statistically significant. Vaccine effectiveness (VE) was either extracted from the publications or calculated as [1-(risk ratio or rate ratio comparing vaccine and control recipients)] × 100. Since heterogeneity between studies was judged to be high with regard to setting, study design, outcome definition and confounders considered in the analysis, no meta-analyses were performed.

## Results

### Search results

By systematic literature search, a total of 1273 publications were identified. Screening of titles and abstracts led to the exclusion of 1074 publications. Of the remaining 199 studies, 22 were found to match our inclusion criteria (see flowchart and list of excluded studies in the Additional file 1: Figure S1 and Table S1). The characteristics of the included studies are listed in Table 1.

**Table 1:** Characteristics of included studies

a.) Studies addressing safety outcomes								
Authors and country	Setting/data sources	Study design/ period	Inclusion (I) and exclusion (E) criteria	Intervention/comparison	Final N/ N potentially eligible/ (%)	N Inter-vention group	N Control group	Outcomes
Munoz et al., 2014; USA [30]	3 National Institutes of Health’s Vaccine Treatment Evaluation Units	RCT, 2008–2012	**I**: Women, 18–45 years of age, with no chronic conditions, a singleton, uncomplicated pregnancy with normal first- or second-trimester screening test results; **E**: Women who received Tdap or any tetanus-containing vaccine within the prior 2 years	Tdap (Adacel®) at 30–32 WG vs. placebo	–	33	15	vaccine-related adverse outcomes; perinatal complications; pertussis illness in infants
Hoang et al., 2016; Vietnam [31]	Primary care	RCT, 2012–2013	**I**: Women, 18–41 years of age, with low risk for complications. **E**: Women with any serious underlying medical condition; febrile illness within 72 h before injection, receipt of TT vaccine in the past month; receipt of Tdap in the past 10 years; receipt of a vaccine, blood product or experimental medicine 4 weeks before or after injection; previous severe reaction to any vaccine	Tdap (Adacel®) at 20–30 WG vs. TT	–	51	48	short-term vaccine-related adverse outcomes; obstetric and perinatal complications
Halperin et al., 2018; Canada [32]	not specified, most likely outpatient hospital care	RCT, 2007–2014	**I**: Healthy, pregnant women 18–45 years of age assessed at ≥30 weeks’ gestation to be at low risk for complications; **E**: Women with high obstetrical risk, history of significant medical disorder or physician-diagnosed pertussis or receipt of Td or Tdap in the last 5 years; sensitivity to Td or Tdap, receipt of blood products or immunoglobulin within 3 months of study entry (except rhesus Ig), or receipt of any vaccines within 2 weeks of study vaccine (except for influenza vaccine).	Tdap (Adacel®) ≥30 WG vs. TT	273/304 (90%)	134	138	acute safety and pregnancy-related outcomes
Berenson et al., 2016; USA [33]	University hospital	RCS, 2012–2014	**I**: Singleton pregnancies delivered ≥27 WG; **E**: Women with < 4 clinic visits during pregnancy	Tdap (vaccine not specified) during pregnancy vs. no Tdap	–	1109	650	obstetric and perinatal complications
DeSilva et al., 2016; USA [34]	7 Vaccine Safety Datalink sites (Analysis of health insurance-based electronic health records)	RCS, 2007–2013	**I**: Singleton live births, women continuously insured from 6 months before LMP through 6 weeks postpartum, with ≥1 outpatient visit(s) during pregnancy. **I** Infants: birth weight and gestational age available; enrolled in health insurance for ≥4 months in first YoL, with ≥1 outpatient visit(s); **E**: Infants with exposures increasing risk for structural birth defects (maternal diabetes or use of teratogenic medications, congenital infections, and chromosomal abnormalities)	Tdap (vaccine not specified) during pregnancy vs. no Tdap	324,463 singleton live births	41,654	282,809	microcephaly and other selected major structural birth defects
DeSilva et al., 2017; USA [35]	7 Vaccine Safety Datalink sites (Analysis of health insurance-based electronic health records)	RCS, 2010–2013	**I**: Singleton live births, women continuously insured from 6 months before LMP through 6 weeks postpartum, with ≥1 outpatient visit(s) during pregnancy. **I** Infants: birth weight and gestational age available; enrolled in health insurance for ≥4 months in first YoL, with ≥1 outpatient visit(s); **E**: Women who received live virus vaccines during pregnancy	Tdap mostly at 27–36 WG (vaccine not specified) vs. no Tdap	197,654 /243,981 (81%) live births	45,008	152,556	obstetric and perinatal complications
Donegan et al., 2014; UK [36]	Primary care practices, (650 primary care general practice databases, 12.5 million patients)	RCS, 2012–2013 Tdap- and 2010–2012 control group	**I** a.) Short-term AE risk: women ≥12 years of age who received pertussis-containing vaccination during pregnancy with ≥28 days of follow-up data after vaccination; **I** b.) Risk throughout pregnancy: women ≥12 years of age with a recorded pregnancy outcome and estimated gestational age with follow-up of at least 44 weeks after the date of the LMP. **I** Historical cohort: women ≥12 years of age with a recorded pregnancy outcome from October 2010 to September 2012 and no record of a vaccine containing pertussis during or after pregnancy	TdaP-IPV (Repevax®) during pregnancy vs. no ap-vaccine	a.): 17,560/ 20,074 (87%); b.): 6185/20,074 (31%)		18,523	obstetric and perinatal complications
Griffin et al., 2018; New Zealand [37]	Nationwide linked administrative health databases	RCS, 2013	**I**: All pregnant women who reached 28–38 WG in 2013; **E** Women: pregnancies < 20 WG or missing maternal or gestational age; **E** Infants: live born babies < 28 WG or BW < 400 g	Tdap (Boostrix®) at 28–38 WG vs. no Tdap	68,550/73,817 (93%)	8178	60,372	obstetric, perinatal and neonatal outcomes
Kharbanda et al., 2014; USA [38]	2 Vaccine Safety Datalink sites (Analysis of health insurance-based electronic health records)	RCS, 2010–2012	**I**: Women 14–49 years of age at delivery with singleton pregnancies ending in live birth, continuously insured from 6 months before LMP through 6 weeks postpartum, ≥1 outpatient visit at an affiliated site and with birth weight and gestational age recorded; **E**: Women who received live virus vaccines during pregnancy or who received Tdap in the 7 days after the estimated pregnancy start date or in the 7 days before delivery; incomplete birth data	Tdap (mainly Adacel®) from 8 days after LMP to 8 days before delivery vs. no Tdap	123,494/300,607 (41%)	26,229	97,265	obstetric and perinatal complications
Kharbanda et al., 2016; USA [39]	Vaccine Safety Datalink sites (Analysis of health insurance-based electronic health records)	RCS, 2007–2013	see Kharbanda, 2014	Tdap (vaccine not specified) during pregnancy vs. no Tdap	427,097/631,256 (68%)	53,885	109,253	acute safety endpoints in 0–42 days after vaccination
Layton et al., 2017; USA [40]	MarketScan Commercial Claims and Encounters (Truven Health Analytics) claims databases of employer-based commercial health care insurance	RCS, 2010–2014	**I**: Women with livebirth or stillbirth deliveries; only first observed pregnancy per women; **E**: Women who delivered at ≤26 WG; women ≤18 years in 13 states with universal childhood immunization policies	Tdap (vaccine not specified) at ≥27 WG; Tdap < 27 WG vs. no Tdap	NR	≥27 WG: 123,780 < 27 WG: 25,037	871,177	acute safety endpoints in 0–42 days after vaccination; obstetrical and perinatal complications
Maertens et al., 2016; Belgium [15]	5 hospitals in Antwerp, Belgium	PCS, 2012–2014	**I**: Women 18–40 years of age with low risk for complications. **E**: Same as Hoang et al.	Tdap (Boostrix®) at 22–33 WG vs. no Tdap	NR	57	42	acute safety outcomes obstetric and perinatal complications
Morgan et al., 2015; USA [41]	Parkland clinic-based pre-natal and obstetrical care centers in Dallas County with centralized electronic medical charting system	RCS, 2013–2014	**I**: All women who delivered at Parkland	Tdap (vaccine not specified) at ≥32 WG vs. no Tdap	NR	7152	226	obstetric and neonatal outcomes
Shakib et al., 2013; USA [42]	Intermountain Healthcare database, Utah	RCS, 2005–2009	**I**: Pregnant women 12–45 years of age and their babies; **E**: Women whose pregnancy start date could not be determined; women who had documentation of Tdap vaccine within 3 days prior to delivery	Tdap (vaccine not specified) at any time during pregnancy vs. no Tdap	162,448	138	552	obstetric and perinatal complications; congenital anomalies, complex chronic conditions in 1st YoL
b.) Studies addressing effectiveness outcomes								
Authors/country	Setting/data source	study design/period	Participants (Inclusion (I) and exclusion (E) criteria)	Intervention/comparator	N	Pertussis cases	control group	outcomes
Amirthalingam et al., 2014, UK [43]	notification data from enhanced surveillance for pertussis cases; and sentinel primary care data (Clinical Practice Research Datalink) for vaccination coverage calculations	RCS; screening method, 2008–2013	**I**: infants < 3 months of age; **E**: unknown maternal vaccination status, vaccination given within 7 days of birth; first primary infant vaccination before 7 days of disease onset	maternal Tdap-IPV (Repevax®) at 28–38 WG vs. no Tdap	NR	71	26,684	laboratory confirmed pertussis at < 2 and < 3 months of age
Amirthalingam et al., 2016, UK [16]	see Amirthalingam et al., 2014, UK [43]	RCS screening method, 2012–2015	see Amirthalingam et al., 2014, UK [43]	maternal Tdap-IPV (Repevax®, Boostrix-Polio®) at 28–38 WG vs. no Tdap	NR	192	72,781	laboratory confirmed pertussis at < 2 and < 3 months of age; pertussis related deaths
Baxter et al., 2017, USA [44]	Kaiser Permanente Northern California (KPNC) medical care data	RCS, 2010–2015	**I**: infants born in KPNC hospitals 2010–2015; full term (> = 37 WG); enrolled in Kaiser health plan by age 4 months; mother continuously enrolled in KPNC health plan; mother born before 1996	maternal Tdap vaccination (Boostrix®, Covaxis®) at least 8 days before birth vs. no Tdap	148,981	17	148,964	laboratory confirmed pertussis at < 2 months of age
Becker-Dreps et al., 2018, USA [45]	commercial insurance claims data	RCS, 2010–2014	**I**: infants </= 18 months of age, delivered between June 2010 and Dec. 2014; First delivery per women; singleton deliveries occurring > 26 WG; **E**: non-continuous insurance enrolment from pregnancy onset until 7 days post-delivery	maternal Tdap vaccination (vaccine not specified) vs. no Tdap	632,825	112	632,713	laboratory confirmed pertussis at < 2 months of age; pertussis-related hospitalization
Bellido-Blasco et al., 2017, Spain [46]	community-based data; cases were identified via computerized mandatory notification system	CCS, 2015–2016	**I**: cases: unvaccinated infants < 3 months old, with confirmed pertussis; controls: three paired controls by age (difference less than 15 days) per case; two controls: same paediatrician/family doctor as case; third control: same maternity clinic as case; controls: unvaccinated	maternal Tdap vaccination (vaccine not specified) vs. no Tdap	88	22	66	laboratory confirmed pertussis at < 3 months of age
Dabrera et al., 2015, England and Wales [47]	community-based data; cases were identified via notification system; controls were 2 infants born consecutively after pertussis case from the same practice	CCS, 2012–2013	**E** infants: aged ≥8 weeks, unknown vaccination status of mother; **E** controls: known clinical or microbiological diagnosis of pertussis	maternal Tdap-IPV (Repevax®) at any time in pregnancy vs. no Tdap	113	58	55	laboratory confirmed pertussis at <2 months ofage
Saul et al., 2017, Australia [48]	cases were identified via notification system; controls: infant born +/−3 days as case in the maternity clinic of the local health district in which the case was notified	CCS, 2015–2016	**E** controls: cough illness within two weeks of the onset of the illness in the matched case	maternal Tdap at ≤2 weeks before birth with a 3-component acellular pertussis vaccine vs. no Tdap	96	48	48	laboratory confirmed pertussis at < 3 months of age; pertussis-related hospitalization
Skoff et al., 2017, USA [49]	cases were identified via surveillance in 6 Emerging Infection Program Network sites; controls were hospital-matched	CCS, 2011–2014	**I**: infants ≥2 days old, residing in the catchment area on their cough onset date, were born in a hospital in their state of residence, were delivered at ≥37 WG, were not adopted or in foster care, and did not live in a residential care facility. **E** controls: pertussis diagnosis prior to the cough onset date of the corresponding case infant	any pertussis-containing vaccine at any time in pregnancy vs. no Tdap	6252	240	535	laboratory confirmed pertussis at <2 months of age; pertussis-related hospitalization

*ap-vaccine* acellular pertussis vaccine, *BW* birth weight, *LMP* last menstrual period, *NR* not reported, *RCT* randomized controlled trial, *RCS* retrospective cohort study, *TT* Tetanus-vaccine, *YoL* year of life, *CCS* case-control-study, *dT5aP-IPV* diphteria-tetanus-5-component-acelluar-pertussis-inactivated-polio-vaccine, *dT3aP-IPV* diphteria-tetanus-3-component-acelluar-pertussis-inactivated-polio-vaccine, *NR* not reported, *WG* weeks of gestation

### Vaccine safety

#### Evidence base and risk of bias

Three RCTs [30–32] and 11 non-randomized studies [15, 33–38, 40–42, 50] from Belgium, United Kingdom, Canada, New Zealand, Vietnam and the USA reported maternal and/or infant safety outcomes (Table 2). Taking into account overlapping study populations of four studies based on the US Vaccine Safety Datalink project [34, 35, 38, 50], data from a total of 1.4 million pregnant women were included, of which 199,846 had received a pertussis-component-containing vaccine during pregnancy. In three RCTs [30–32] and one non-randomized study [42] the pertussis-containing vaccine used was Adacel®, whereas in four other studies it was Boostrix® [15, 37, 52, 53] and in the British study [36] it was Repevax®. In most studies from the US [33–35, 38, 40, 41, 50] the vaccine used was not specified.

**Table 2:** Safety outcomes

Study	Outcome definition	Design	Intervention/comparison group	WG at vaccination: mean (range)	Tdap-vaccinated group			Control group			Unadjusted estimate (95%CI)	Adjusted estimate (95% CI)
					N	n_cases_	%	N	n_cases_	%		
**Fever**												
Hoang et al., 2016 [31]	Self-reported fever without time limit	RCT	Tdap/TT	25.8 (18–36)	52	1	1.9	48	0	0.0	NR	NR
Munoz, 2014 [30]	Oral temperature of ≥38° Celsius during 7 days after Tdap vaccination	RCT	Tdap/placebo	30–32	33	1	3.0	15	0	0.0	NR	NR
Maertens et al., 2016 [15]	Fever	PCS	Tdap/no Tdap	28.6 (22–33)	57	1	1.8	42	0	0.0	NR	NR
Kharbanda et al., 2016 [39]	Medically attended fever during 3 days after Tdap vaccination	RCS	Tdap/no Tdap	81.8% ≥20	53,885	15	0.03	109,253	6	0.006	2.16 (1.65–2.83)^a^	NR
**Stillbirth**												
Hoang et al., 2016 [31]	Stillbirth	RCT	Tdap/TT	25.8 (18–36)	52	0	0.0	51	1	2.0	NR	NR
Berenson et al., 2016 [33]	Stillbirth	RCS	Tdap/no Tdap	30.3 (1–40)	650	0	0.0	1109	1	0.1	NR	NR
Donegan et al., 2014 [36]	Intrauterine death after 24 WG within 14 days of vaccination	RCS	Tdap-IPV/no Tdap	31 (29–35)	13,371	5	0.0	13,371	7.2 (expected)	0.1	0.69 (0.23–1.62)	NR
	Intrauterine death after 24 WG from vaccination to delivery			33 (30–36)	6185	12	0.2	18,523	42	0.3	0.85 (0.44–1.61)	NR
Morgan et al., 2015 [41]	Stillbirth	RCS	Tdap ≥32 WG/no Tdap	≥32	7152	25	0.3	226	1	0.4	0.79 (0.11–5.85)^b^	NR
Shakib et al., 2013 [42]	Stillbirth	RCC	Tdap 3–280 days prepartal/no Tdap	87 (63%) 1st, 24 (17%) 2nd, 27 (20%) 3rd trimester	138	0	0.0	552	5	0.9%	0.36 (0.02–6.54)^b^	NR
**Neonatal death**												
Morgan et al., 2015 [41]	Not defined	RCS	Tdap ≥32 WG/no Tdap	≥32 WG	7152	2	0.028	226	0	0	0.16 (0.01–3.31)^b^	NR
Donegan et al., 2014 [36]	Neonatal death within 7 days of delivery	RCS	Tdap-IPV/no Tdap	33 (30–36)	6185	2	0.032	18,523	6	0.032	1.00 (0.20–4.95)	NR
**Preterm birth**												
Berenson et al., 2016 [33]	< 37 WG	RCS	Tdap/no Tdap	30.3 (1–40)	1109	58	5.2	650	59	9.1	0.77 (0.64–0.93)^a^	0.68 (0.45–1.03)
Kharbanda et al., 2014 [38]	< 37 WG	RCS	Tdap in any WG/no Tdap	2.014 (7.7%) 1st, 10.936 (41.7%) 2nd, 13.280 (50.6%) 3rd trimester	26,229	1.527	6.3	97,265	7544	7.8	1.01 (0.95–1.06)	1.03 (0.97–1.09)
			Tdap 27–36 WG/no Tdap		11,351	602	5.3	97,265	7544	7.8	0.88 (0.81–0.96)	0.88 (0.80–0.95)
Layton et al., 2017 [40]	Not defined; presumably < 37 WG	RCS	Tdap/no Tdap	< 27	25,037	2.593	10.4	871,177	66,968	7.7	1.37 (1.32–1.43)^a^	NR
				≥27	123,780	6.154	5.0	871,177	66,968	7.7	0.66 (0.64–0.68)^a^	NR
Shakib et al., 2013 [42]	< 37 WG	RCC	Tdap 3–280 days antepartum/no Tdap	87 (63%) 1st, 24 (17%) 2nd, 27 (20%) 3rd trimester	134	8	6.0	505	38	7.5	0.78 (0.36–1.71)^b^	NR
Munoz et al., 2014 [30]	< 37 WG	RCT	Tdap/placebo	30–32	33	3	9.1	15	1	6.7	1.50 (0.14–15.67)^b^	NR
Griffin et al., 2018 [37]	Premature birth ICD-10-AM O60.1.3; birth < 37 WG	RCS	Tdap 28–38 WG/no Tdap	33. IQR: 30–35	8178	297	3.6	60,372	2829	4.7	0.74 (0.66–0.84)	0.72 (0.63–0.83)
Morgan et al., 2015 [41]	< 37 WG	RCS	Tdap at ≥32 WG/no Tdap	≥32	7152	427	5.9	226	27	11.9	0.47 (0.31–0.71)^b^	NR
Hoang et al., 2016 [31]	Not defined	RCT	Tdap/ TT	25.8 (18–36)	52	0	0.0	51	1	2.0	NR	NR
DeSilva et al., 2017 [35]	< 34 WG	RCS	Tdap in any WG/no Tdap	51% 27–36	45,008	426	0.9	152,556	2711	1.8	0.59 (0.54–0.65)^a^	NR
Halperin et al., 2018 [32]	MedDRA version 19.0 - definition	RCT	Tdap/Td	34.5 (32.6–35.6)	134	2	1.5	138	1	0.7	2.06 (0.19–22.45)	NR
**Low birth weight**												
Berenson et al., 2016 [33]	LBW: < 2500 g	RCS	Tdap/no Tdap	30.3 (1–40)	1109	61	5.5	650	59	9.1	NR	0.76 (0.51–1.14)
	VLBW: < 1500 g				1109	2	1.1	650	12	0.3	NR	0.24 (0.05–1.20)
Griffin et al., 2018 [37]	Intrauterine growths retardation (ICD10-AM O36.5)	RCS	Tdap 28–38 WG/no Tdap	33. IQR: 30–35	8178	401	4.9	60,372	2916	4.8	0.94 (0.84–1.04)	0.92 (0.82–1.04)
Donegan et al., 2014 [36]	Intrauterine growths retardation/LBW < 2500 g	RCS	Tdap-IPV/no Tdap	33 (30–36)	6185	126	2.0	18,523	311	1.7	1.20 (0.98–1.48)	NR
**Neonatal sepsis**												
Layton et al., 2017 [40]	Sepsis in the newborn during 30 days after birth	RCS	Tdap/no Tdap	< 27 WG	16,322	394	2.41	543,906	13,187	2.27	1.07 (0.97–1.18)	0.89 (0.81–0.99)^c^ 0.91 (0.81–1.02)^d^
				≥27 WG	80,217	1.774	1.84	543,906	13,187	2.27	0.81 (0.77–0.85)	0.83 (0.79–0.88)^c^ 0.89 (0.84–0.94)^d^
**Admission to newborn intensive care unit**												
Layton et al., 2017 [40]	Admission to newborn intensive care unit during 30 days after birth	RCS	Tdap/no Tdap	< 27 WG	16,322	1.458	8.93	543,906	42,904	7.39	1.22 (1.16. 1.29)	0.93 (0.88–0.98)^c^ 0.95 (0.89–.01)^d^
				≥27 WG	80,217	6996	7.25	543,906	42,904	7.39	0.98 (0.96–1.01)	0.97 (0.95–1.00)^c^ 1.00 (0.97–1.03)^d^
Berenson et al., 2016 [33]	Not defined	RCS	Tdap/no Tdap	30.3 (1–40)	1109	103	9.3	650	86	13.2	NR	0.78 (0.56–1.08)
**Preeclampsia and eclampsia**												
Griffin et al., 2018 [37]	Hypertension (ICD10-AM O13-O16)	RCS	Tdap in 28–38 WG/no Tdap	33. IQR: 30–35	8178	262	3.2	60.372	1484	2.5	1.20 (1.05–1.37)	1.02 (0.88–1.19)
	Preeclampsia (ICD10-AM O14.09)					133	1.6		1007	2.5	0.91 (0.75–1.09)	0.85 (0.69–1.04)
	Severe preeclampsia (ICD10-AM O14.1)					26	0.3		271	0.4	0.67 (0.45–1.00)	0.61 (0.39–0.94)
Layton et al., 2017 [40]	Preeclampsia/eclampsia (642.4x-642.8x)ICD-9 codes 624.xx during 7 days pre- and up to 30 days postpartum	RCS	Tdap/no Tdap	< 27	25,037	1.096	4.38	871,177	40,930	4.4	1.00 (0.94–1.06)	0.99 (0.93–1.05)^c^ 1.05 (0.99–1.12)^d^
				≥27	123,780	5.248	4.24	871,177	40,930	4.4	0.96 (0.94–0.99)	0.90 (0.87–0.93)^c^ 0.96 (0.94–0.99)^d^
Kharbanda et al., 2014 [38]	Gestational hypertension (ICD642.3x). Hypertension in pregnancy (ICD642.9) Preeclampsia or eclampsia (ICD642.4x-642.8x); onset ≥20 WG	RCS	Tdap< 20 WG/no Tdap	2.014 (7.7%) 1st, 10.936 (41.7%) 2nd, 13.280 (50.6%) 3rd trimester	6083	497	8.2	97,265	7736	8.0	1.03 (0.94–1.12)	1.09 (0.99–1.20)
Donegan et al., 2014 [36]	Not defined; clinical diagnoses during pregnancy from primary care general practice databases	RCS	Tdap-IPV/no Tdap	33 (30–36)	6185	22	0.4	18,523	54	0.3	1.22 (0.74–2.01)	NR
Halperin et al., 2018 [32]	Preeclampsia (MedDRA version 19.0- definition)	RCT	Tdap/Td	34.5 (32.6–35.6)	134	1	0.7	138	2	1.4	0.51 (0.05–5.61)	NR
Maertens et al., 2016 [15]	Preeclampsia	PCS	Tdap/no Tdap	28.6 (22–33)	57	4	7.0	41	1	2.4	1.40 (0.88–2.25)^a^	NR
	Hypertension				57	2	3.5	41	1	2.4	1.15 (0.51–2.61)^a^	NR
	Preeclampsia or hypertension				57	6	10.5	41	2	4.9	1.32 (0.85–2.05)^a^	NR
**Malformations**												
Hoang et al., 2016 [31]	Not defined. Not reported in results section	RCT	Tdap/TT	25.8 (18–36)	52	0	0.0	48	0	0.0	NR	NR
Munoz et al., 2014 [30]	Not defined	RCT	Tdap/placebo	30–32	33	1	3.0	15	2	13.3	0.2 (0.02–2.44)^b^	NR
Berenson et al., 2016 [33]	Ten most commonly encountered birth defects reported by the Centers for Disease Control and Prevention	RCS	Tdap/no Tdap	30.3 (1–40)	1109	18	1.6	650	15	2.3	NR	0.80 (0.38–1.67)
DeSilva et al., 2016 [34]	Diagnostic codes for any structural birth defect, diagnosed during first YOL	RCS	Tdap/no Tdap	any WG	41,654	2816	6.8	282,809	17,422	6.2	1.09 (1.05–1.14)	0.98 (0.94–1.03)
				27–36 (50%)	20,568	1435	7.0	120,097	8367	7.0	1.00 (0.95–1.06)	1.02 (0.96–1.08)
	Diagnostic codes for selected major structural birth defects: such as spina bifida (741.0x and 741.9x); encephalocele, etc., diagnosed during first YOL			any WG	41,654	717	1.7	282,809	4521	1.6	1.08 (1.00–1.17)	1.06 (0.98–1.16)
				27–36 (50%)	20,568	356	1.7	120,097	1920	1.60	1.08 (0.97–1.21)	1.09 (0.97–1.23)
	Diagnostic codes for microcephaly, diagnosed during first YOL			any WG	41,654	38	0.09	282,809	348	0.12	0.74 (0.53–1.04)	0.86 (0.60–1.24)
				27–36 WG (50%)	20,568	21	0.10	120,097	146	0.12	0.84 (0.53–1.33)	1.01 (0.63–1.61)
Maertens et al., 2016 [15]	Not defined	RCS	Tdap/no Tdap	28.6 (22–33)	57	0	0.0	42	0		NR	NR
Morgan et al., 2015 [41]	Major malformations	RCS	Tdap ≥32 WG/no Tdap	≥32 WG	7152	84	1.2	226	3	1.3	0.88 (0.28–2.82)^b^	NR
**Chorioamnionitis**												
Berenson et al., 2016 [33]	Physician’s diagnosis in medical file AND fever ≥38 °C AND ≥1 of the following symptoms: uterine tenderness, malodorous vaginal discharge or maternal leucocytosis	RCS	Tdap in any WG/no Tdap	30.3 (1–40)	1.109	39	3.5	650	14	2.2	NR	1.53 (0.80–2.90)
Kharbanda et al., 2014 [38]	ICD-9 code 658.41 as diagnosis during stay in birth clinic	RCS	Tdap in any WG/no Tdap	2014 (7.7%) 1st, 10,936 (41.7%) 2nd 13,280 (50.6%) 3rd trimester	26,229	1596	6.1	97,265	5329	5.5	1.11 (1.05–1.17)	1.19 (1.13–1.26)
			Tdap during 27–36 WG/no Tdap		11,351	637	5.6	97,265	5329	5.5	1.02 (0.95–1.11)	1.11 (1.03–1.21)
Layton et al., 2017 [40]	ICD-9762.7. 658.4. 658.4x coded for mother or newborn during stay in birth clinic	RCS	Tdap/no Tdap	< 27	25,037	984	3.93	871,177	25,149	2.7	1.45 (1.37–1.55)	1.23 (1.16–1.31)^c^ 1.19 (1.11–1.28)^d^
				≥27	123,780	4529	3.66	871,177	25,149	2.7	1.35 (1.31–1.40)	1.14 (1.10–1.18)^c^ 1.11 (1.07–1.15)^d^
DeSilva et al., 2017 [35]	ICD-9 code 658.41 from maternal medical file during stay in birth clinic	RCS	Tdap at any WG/no Tdap	51% 27–36	45,008	2.883	6.4	152,556	7970	5.2	NR	1.23 (1.17–1.28)
			Tdap during 27–36 WG/no Tdap		22,772	1430	6.3	133,882	7109	5.3	NR	1.20 (1.14–1.28)
Griffin et al., 2018 [37]	ICD-10-AM O41.1	RCS	Tdap during 28–38 WG/no Tdap	33. IQR: 30–35	8178	26	0.3	60,372	198	0.3	0.89 (0.59–1.34)	1.10 (0.70–1.75)
Morgan et al., 2015 [41]	Not defined. Extracted from data base with information on pregnancy, birth and newborn	RCS	Tdap ≥32 WG/no Tdap	≥32	7152	421	5.9	226	9	4.0	1.51 (0.77–2.96)^b^	NR

*ICD* international classification of disease, *LBW* low birth weight, MedDRA Medical Dictionary for Regulatory Activities, *NR* not reported, *PCS* prospective cohort study, *RCS* retrospective cohort study *RCT* randomized controlled trial, *Tdap* tetanus-diphtheria-acellular pertussis vaccine, *VLBW* very low birth weight, *WG* week of gestation, *YOL* year of life

^a^calculated using STATA (csi command; (Cornfield method for the calculation of confidence intervals)

^b^from McMillan et al., 2017

^c^adjusted for: maternal age, year of birth, maternal hospital stays and ambulatory consultations, other insured children, US region, living in a statistical metropolitan area of the USA, undergone prenatal obstetric blood test, undergone sonography, hypertension, diabetes, gestational diabetes, impaired kidney function, lupus, taking antihypertensive medication, taking antidiabetic medication, taking antidepressive therapy, taking antibiotics

^d^Propensity score (PS) estimated for Tdap receipt by logistic regression and use of maternal characteristics and transformed into „stabilized inverse probability of treatment weights (IPTW)“

In most studies, women who had received tetanus-diphtheria-acellular pertussis (Tdap) vaccines (Adacel® or Boostrix®) or Tdap-IPV-vaccines (Repevax®), were compared to women, who were either unvaccinated or had received placebo. In two RCTs, the comparison group was vaccinated with a tetanus-toxoid-containing vaccine [31, 32].

Risk of bias (RoB) was judged high for one [31] and low for two RCTs [30, 32] (Additional file 1: Table S2). Vaccine administrators were not blinded in the trials by Halperin et al. [32] and Munoz et al. [30], but we considered this to be unlikely to have influenced the outcomes “prematurity“, “pre-eclampsia/eclampsia“, “fever” and “malformations” in these studies.

Of the 11 non-randomized studies, we judged eight as having a serious RoB and three studies to show a critical RoB (Additional file 1: Table S2). The main reasons for these classifications were confounding, selection bias, and imprecise outcome assessment. Residual confounding could not be excluded in any of the studies. In addition, a likely healthy vaccinee bias was observed in most studies. Preexisting comorbidities (e.g. arterial hypertension [34, 38, 41], heart disease [38], diabetes [34, 41], pulmonary disease [34, 38]) and referral to high-risk obstetrics clinics [41] were more frequent in non-vaccinated women than in vaccinated women. In addition, health care utilization differed between vaccinated and non-vaccinated women (e.g. higher uptake of influenza vaccination [33, 37, 40, 54] and ultrasound examinations [40, 54] during pregnancy among Tdap vaccinated women). In several studies, Tdap-vaccinated women showed indications for better uptake or earlier start of prenatal care [33, 38, 40, 41]. Frequently, these results were statistically significant [33, 38, 41]. Healthy vaccinee bias might have shifted estimates towards more favorable outcomes in vaccinated women and their infants. Moreover, with respect to preterm birth, immortal time bias could also have influenced the results.

In two studies [38, 39], a large proportion (74 and 79% respectively) of eligible study participants was excluded from analysis, e.g., women with irregular health insurance status or with a history of multiple pregnancies, stillbirth or premature birth. This might have limited generalizability of study results. In addition, exclusion of pregnancies ending in stillbirth or abortion might have resulted in selection bias with regard to potentially associated outcomes, like congenital malformations.

Five studies were based on commercial health data bases using ICD codes [34, 35, 38–40]. Coding for commercial reasons, such as insurance claims, might be prone to favoring more severe diagnoses. This might be a relevant source of bias for some outcomes, such as chorioamnionitis, but not for others, like admission to NICU. In studies, which were based on medical records [33, 36], no standardized case definitions were used, such as those developed by the Brighton Collaboration [55]. Here, potential misclassification was likely to be non-differential.

#### Fever

Rates of fever after Tdap vaccination in pregnancy were assessed in four studies [15, 30, 31, 50]. The definition of fever varied considerably across studies (Table 2). Overall, fever following immunization was reported in 0.03 to 3% of pregnant women and occurred more frequently in Tdap-vaccinated women than in control women.

#### Stillbirth

One RCT [31] and four non-randomized studies [33, 36, 41, 42] assessed stillbirths. None of these studies reported an increased risk in Tdap-vaccinated women.

#### Neonatal deaths

Two non-randomized studies reported on neonatal death [36, 41]. A few cases were observed but there was no significant association with Tdap vaccination during pregnancy.

#### Preterm birth

Ten of the included studies reported on preterm birth, mostly defined as gestational age <37 weeks. In the three RCTs [30–32], only a few preterm births were observed without differences between vaccinated and control mothers. In seven non-randomized studies [33, 35, 37, 38, 40–42], risk of preterm birth was higher in unvaccinated than in Tdap-vaccinated women. In one of these studies [40], compared to no vaccination, Tdap-vaccination at 27–36 weeks gestation was associated with a decreased risk of preterm birth, whereas earlier vaccination (before 27 weeks) was associated with an increased risk (see Table 2).

#### Low birth weight

Low birth weight (LBW; < 2500 g) or very low birth weight (VLBW; < 1500 g) were assessed in three studies. Donegan et al. [36] reported on intrauterine growth retardation/LBW, Berensen et al. [33] assessed LBW and VLBW separately. Neither study reported an association between Tdap-vaccination during pregnancy and low birth weight. Griffin et al. [37] assessed the outcome “fetal growth restriction” and also found no association with Tdap-vaccination during pregnancy.

#### Congenital malformations

Definition and recording of congenital malformations varied considerably across the seven studies which reported on this outcome. Hoang et al. [31] did not detect any malformations within 30 days after birth, while Munoz et al. [31] observed one case of pyelectasia in an infant of a Tdap-vaccinated mother and two cases of cardiac malformation in infants whose mothers had not been vaccinated. In five non-randomized studies [15, 33, 34, 41, 42], the authors observed no association between Tdap-vaccination during pregnancy and malformations including infants of mothers who were vaccinated during the first trimester in the study by DeSilva et al. [34].

#### Neonatal septicaemia

Only one non-randomized study [40] reported on neonatal sepsis, but did not distinguish between early- and late-onset sepsis. Newborns of Tdap-vaccinated mothers were at lower risk of septicemia than newborns of unvaccinated mothers.

#### Admission to NICU

Two non-randomized studies reported on NICU admission [33, 40]. Risk of NICU admission was lower in newborns of vaccinated mothers than in those of unvaccinated mothers in both studies.

#### Pre-eclampsia and eclampsia

The six non-randomized studies that reported on pre-eclampsia and eclampsia used heterogeneous definitions for this outcome (see Table 2). Layton et al. [40] observed a slightly decreased risk for pre-eclampsia and eclampsia in women who had been vaccinated after 26 weeks of gestation, compared to unvaccinated women (RR: 0.96; CI: 0.94–0.99). Similar findings were obtained by Griffin et al. [37] for severe pre-eclampsia (RR: 0.61; CI: 0.39–0.94). In the remaining four studies [15, 32, 36, 38], no association between Tdap-vaccination and pre-eclampsia or eclampsia was observed.

#### Chorioamnionitis

Chorioamnionitis was investigated in six non-randomized studies [33, 35, 37, 38, 40, 41]. All of them reported an increased risk of chorioamnionitis in women who had received Tdap-vaccination during pregnancy (Table 2). Of those, two studies investigated Tdap given at any week of gestation, while in the remaining four studies Tdap vaccination was performed in the third trimester (> = 27 to > = 32 weeks of gestation). In the four largest studies comprising 8178 to 123,780 vaccinated women, the outcome was defined using ICD-codes [35, 37, 38, 40]. One study [41] did not report the outcome definition. Only Berenson et al. [33] used a clinical case definition for identifying chorioamnionitis in electronic medical records. In the six studies, risk ratios ranged from 1.04 (95%CI: 0.98–1.11) to 1.53 (95%CI: 0.80–2.90). Size of risk estimates was unrelated to time point of Tdap vaccination during pregnancy. Estimates were statistically significant in three studies [35, 38, 40].

In order to minimize the influence of health seeking behavior, Layton et al. [40] conducted a subgroup analysis restricting the cohort to pregnant women who were vaccinated against influenza. In this subgroup, the association between Tdap-vaccination and chorioamnionitis was weaker (adjusted RR: 1.09; 95%CI: 1.03–1.15) than in the full cohort analysis (adjusted RR: 1.14; 95%CI: 1.10–1.18). When propensity score adjustment was used, the estimate was no longer statistically significant.

### Vaccine effectiveness

#### Evidence base and risk of bias

Eight studies fulfilled the inclusion criteria for the assessment of vaccine effectiveness (VE), including four cohort studies [16, 43–45] and four case-control-studies [46–49] (Table 2). The populations of the studies by Amirthalingam et al. [43] and Dabrera et al. [47] were included in the study by Amirthalingam et al. [16] (personal communication, Gavin Dabrera, October 17th, 2018). Taking into account this overlap, a total of 855,546 mother-infant-pairs were included in the studies, including 682 pertussis cases in infants < 3 months (thereof 257 < 2 months) of age and 854,864 non-cases. Mothers of 84 cases (12%) and 205,919 non-cases (24%) were vaccinated.

Five [16, 43, 45, 47, 49] of the eight studies were judged as having a serious RoB, while three studies had a moderate RoB [44, 46, 48]. The main reasons for these classifications were selection bias, and imprecise outcome definitions. Using the screening method, Amirthalingam et al. [16, 43] could not adjust estimates for confounders other than age and time period. Residual confounding was judged likely to be present in the other studies as well, as adjustment for potential confounders was limited (see below). Therefore, and since there was evidence that vaccinated women had a more favorable health profile than those not vaccinated (e.g., uptake of influenza vaccination and ultrasound examination during pregnancy were more frequent in Tdap vaccinated women [45], smokers were more frequent in households of non-vaccinated women [46, 48]), a “healthy vaccinee bias” appeared likely, suggesting that VE based on these studies might be overestimated.

The study by Skoff et al. [49] was judged as having a serious RoB because two-thirds of the initially identified study population was excluded and evidence for selection bias was found (the level of education and geographical distribution of study participants and excluded population differed significantly). The most common reasons for exclusion were non-reachability and missing consent to participate. We judged the most recent cohort study from the USA by Becker-Dreps et al. [45] as having a serious RoB because no clear case definition based on laboratory criteria was reported and the proportion of lab-confirmed cases in the subgroups was unknown.

#### Pertussis in infants 0–3 months of age

Three cohort [16, 43, 44] and two case-control studies [47, 49] reported on the effectiveness of Tdap vaccination in pregnancy to prevent pertussis in infants 0–2 months of age (Table 3). In all studies except for the one by Skoff et al. [49], only laboratory confirmed cases were included. Skoff et al. [49] also included cases with an epidemiological link or with a clinical picture of pertussis in their analysis (6% of all cases). Confounder-adjusted VE estimates in these five studies ranged from 78 to 93%.

**Table 3:** Vaccine effectiveness outcomes

Study	Design	Intervention/ control	Study population	Pertussis cases			Non-cases /controls			unadjusted effect estimate (95%CI)	adjusted effect estimate (95%CI)
				N	Cases with vaccinated mothers		N	Non-cases with vaccinated mothers			
					n	%		n	%		
**Prevention of laboratory confirmed pertussis in infants aged 0–2 months**											
Amirthalingam et al., 2014 [43]	CS, screening method	dTaP-IPV/no vaccination	26,684	71	11	14			61	VE = 90% (82–95)	NR
Amirthalingam et al., 2016 [16]	CS, screening method	dTaP-IPV/no vaccination	72,781	192	31	16			64	VE = 90% (86–93)	NR
Baxter et al., 2017 [44]	CS	Tdap/no vaccination	148,981	17	1	6	148,964	68,167	46	IRR = 0.08 (0.00–0.43); VE = 87%	91% (20–99)
Dabrera et al., 2015 [47]	CCS	dTaP-IPV/no vaccination	113	58	10	17	55	39	71	OR: 0.09 (0.03–0.23) VE = 91% (77–97)	93% (81–97)
Skoff et al., 2017 [49]	CCS	Tdap/no vaccination	775	240	17	7	535	90	17	VE = 62%	78% (48–90)
Becker-Dreps et al., 2018 [45]	CS	Tdap/no vaccination	632,825	112	7	0.01	632,713	90,438	0.02	HR: 0.33 (0.12–0.90); VE: 67%	HR: 0.54 (0.19–1.59); VE: 46%
**Prevention of laboratory confirmed pertussis in infants aged 0–3 months**											
Amirthalingam et al., 2014 [43]	CS, screening method	dTaP-IPV/no vaccination	26,684	82	12	15			62	VE = 91% (84–95)	NR
Amirthalingam et al., 2016 [16]	CS, screening method	dTaP-IPV/no vaccination	72,781	243	35	14			65	VE = 91% (88–94)	NR
Bellido-Blasco et al., 2017 [46]	CCS	Tdap/no vaccination	88	22	5	23	66	41	62	OR: 0.08 (0.017–0.371)	91% (57–98)
Saul et al., 2017 [48]	CCS	Tdap/no vaccination	96	48	19	40	48	33	69	OR: 0.36 = VE: 64%	69% (13–89)
**Prevention of hospitalization due to laboratory confirmed pertussis in infants aged 0–2 and 0–3 months, respectively (percentage of lab-confirmed cases unclear in the study by Becker-Dreps et al.)**											
Saul et al., 2017 [48]	CCS (age ≤ 3 months)	Tdap/no vaccination	74	37			37			OR: 0.16 (0.05–0.53)	OR: 0.06 (0.01–0.41); VE: 94% (59–99)
Skoff et al., 2017 [49]	CCS (age ≤ 2 months)	Tdap/no vaccination	6252	157			535			NR	2nd trimester: 91% (25–99) 3rd trimester: 91% (65–97)
Becker-Dreps et al., 2018 [45]	CS (age ≤ 2 months)	Tdap/no vaccination	632,825	80	4	0.00	632,745	90,441	0.01	HR = 0.23 (0.06–0.96) VE: 77%	HR = 0.34 (0.08–1.50) VE: 66%
Prevention of death due to laboratory confirmed pertussis in infants aged 0–3 months											
Amirthalingam et al., 2016 [16]	CS, screening method	dTaP-IPV/ No vaccination	243	11	1	9	232	158	68	VE = 95% (79–100)	NR

*CCS* case-control-study, *CS* cohort study, *HR* hazard ratio, *IRR* incident rate ratio, *NR* not reported, *OR* odds ratio, *VE* vaccine effectiveness, *Tdap* tetanus-diphtheria-acellular pertussis vaccine, *Tdap-IPV* tetanus-diphtheria-acellular pertussis-inactivated polio-vaccine

Four studies - the aforementioned two UK-based cohort studies by Amirthalingam et al. [16, 43] and two case-control studies from Spain and Australia [46, 48] - reported vaccine effectiveness estimates for prevention of laboratory-confirmed pertussis in infants 0–3 months of age between 69 und 91% (Table 3). The age and time-period-adjusted point estimates of both studies by Amirthalingam et al. [16, 43] and the adjusted point estimate of the study by Bellido-Blasco et al. [46] were all 91%. Bellido-Blasco et al. [46] adjusted their analysis for breastfeeding, maternal level of education and presence of other children in the household. The VE estimate of Saul et al. [48] of 69% (95%CI: 13–89%) was adjusted for breastfeeding, household size and gestational age.

#### Pertussis-related hospitalization in infants 0–3 months of age

The confounder-adjusted VE estimates for the prevention of hospitalization due to pertussis in infants were 91 and 94% in two case-control studies from the US (California) [49] and Australia [48], respectively. Saul et al. [48] included infants ≤3 months of age, whereas Skoff et al. [49] focused on infants ≤2 months of age. The case definition used by Saul et al. [49] included only laboratory confirmed cases. Skoff et al. [49] included 6% clinical cases without laboratory confirmation (Table 3).

In the recent US American cohort study [45] which reported a VE of 66% for the prevention of hospitalization due to pertussis in infants ≤2 months of age, the percentage of laboratory confirmed cases was unclear.

#### Pertussis-related deaths in infants aged 0–3 months

One cohort study from the United Kingdom [16] reported an effectiveness of 95% for Tdap vaccination in pregnancy for the prevention of death due to laboratory confirmed pertussis in infants 0–3 months of age (Table 3). Due to the use of the screening method, only an unadjusted effect estimate could be reported, which was based on 11 cases.

### Quality of evidence

Regarding safety outcomes, quality of evidence according to GRADE was judged as low to very low. Reasons for downgrading were serious to critical risk of bias and imprecision. The evidence related to three of four effectiveness outcomes (pertussis < 2 months of age, pertussis < 3 months of age, pertussis-related death) was also downgraded to low quality due to moderate to serious risk of bias and inconsistency. Evidence quality for the remaining effectiveness outcome (hospitalization) was assessed as moderate due to risk of bias (see the GRADE evidence profile in Additional file 1: Table S3 for details).

## Discussion

In this comprehensive systematic review, we evaluated the safety and effectiveness of acellular pertussis vaccination during pregnancy. Using data from more than 1.4 million pregnancies, we found similar risks for all pre-specified safety outcomes in vaccinated and unvaccinated women and their infants except for two: slightly increased relative risks were detected for post-vaccination fever and chorioamnionitis at the time of delivery after Tdap vaccination in all studies reporting these outcomes. The risk increase was significant in one study reporting on maternal fever [50] and in three studies reporting on chorioamnionitis [35, 38, 40]. High effectiveness of Tdap-vaccination during pregnancy in preventing pertussis and related complications in the newborn and young infant was observed in all studies. Using GRADE methodology, the overall quality of evidence was rated as moderate to very low, depending on the outcome category.

Fever is a well-known adverse event after Tdap vaccination occurring at similar [30, 40, 56] or lower [40, 57] frequency in pregnant compared to non-pregnant women. The variation in the reported rates in the four included studies can be explained by the differences in the outcome assessments and fever definitions (self-reported versus measured versus medically attended fever). Based on the largest cohort study’s estimate [50], we calculated that about 6 additional cases of fever per 100,000 pregnant women would occur after Tdap vaccination, as compared to no vaccination (using the difference of absolute risks in vaccinated versus non-vaccinated women).

Six studies reported a small increased relative risk of chorioamnionitis after Tdap vaccination. Three studies showed a significantly increased risk. All three were performed in the USA and had very large numbers of participants. They used “presence of respective ICD-9 codes in electronic patient data” as definition of chorioamnionitis [35, 38, 40].

Chorioamnionitis is an inflammation of the fetal membranes, the amniotic cavity including its fluid and of the placenta, predominantly due to ascending bacterial infections [58, 59]. It may occur at any time during pregnancy or delivery and may be preceded [59] or followed [58] by premature rupture of membranes. Chorioamnionitis is defined by either clinical features [58, 60], microbiological findings, histopathological signs [59] or a combination of these. Clinically relevant chorioamnionitis is a frequent cause of preterm birth and may lead to neonatal sepsis [59]. In the USA, a clinical and/or histologically proven chorioamnionitis is diagnosed in 40–70% of preterm deliveries and in 1–13% of term deliveries [59]. From an immunological perspective, it appears plausible that vaccination can trigger an inflammatory process in pregnancy. During the course of pregnancy, the immune system of the expectant mother undergoes changes in its actiity, ranging from local inflammation that accompanies the tumor-like implantation of the fetus (first trimester) to the predominance of immune tolerance (second trimester) and ending up with inflammatory signals that lead to the induction of labor (third trimester) [61]. Tdap vaccination is only one of multiple activating stimuli to the maternal immune system during pregnancy. To our knowledge, so far no studies on pregnant animals have been published that examined the consequences of immune stimulation by vaccines for the outcome of pregnancy.

If chorioamnionitis were causally related to Tdap-vaccination in the studies that were included in this review, for instance through some as yet unknown immunologic mechanism, we would expect increased risks of preterm birth or sepsis in infants of Tdap-vaccinated women. However, in seven studies [33, 35, 37, 38, 40–42], including those reporting a significant association between Tdap vaccination and chorioamnionitis, rates of preterm birth were even lower in Tdap-vaccinated than in unvaccinated women. Furthermore, in three studies the risk of NICU admission [33, 40] and sepsis [18] was also lower in infants of Tdap-vaccinated mothers as compared to infants of unvaccinated mothers, including one of the studies that reported an increased risk of chorioamnionitis [33, 40].

Hypothesizing that ICD-codes derived from electronic databases might not correctly reflect the clinical, microbiologic or histopathologic diagnosis of chorioamnionitis, Kharbanda et al. [38] validated the diagnosis by subgroup analysis. They randomly selected a validation sample of 220 women with hospital discharge ICD-codes for chorioamnionitis from electronic health charts. “Probable chorioamnionitis” was defined as the presence of ICD-9-Code 658.41 in combination with at least two clinical signs (maternal or fetal tachycardia, uterine tenderness, purulent or foul smelling amniotic fluid). Based on this definition the authors calculated that the positive predictive value (PPV) of the ICD-code for “probable chorioamnionitis” was 50%. When applying this PPV to the whole study population the association between Tdap vaccination in pregnancy and chorioamnionitis remained statistically significant. However, for the subgroup of women vaccinated between 27 and 36 weeks gestation, the association was no longer statistically significant (*p* = 0.07).

Based on our analyses healthy vaccinee bias is a likely confounding factor in most studies, irrespective of study design. This could be an explanation for lower risks for potential sequelae of chorioamnionitis and for the increased frequency of diagnosing and coding chorioamnionitis as a consequence of better ante- and perinatal care including a more careful surveillance of vaccinated women (detection bias).

One possible explanation for the association between Tdap vaccination in pregnancy and chorioamnionitis might be confounding with epidural anesthesia. In a secondary analysis of data from a randomized trial, Abramovici et al. [62] reported a statistical association between use of epidural anesthesia and chorioamnionitis defined as the presence of fever and a physician’s diagnosis warranting antibiotics. In this study, placental histopathologic examination revealed acute inflammation in 70% of those cases of clinical chorioamnionitis in which placental pathology was available for review (64%). Placental culture was not performed. In the cohort study by Maertens et al. [15], 70% of vaccinated versus 57% of unvaccinated women received epidural anesthesia, and in the subgroup analysis of Kharbanda et al. 95% of the 220 women with ICD-9 codes for chorioamnionitis had received an epidural anesthesia and 91% antibiotic treatment (all 220 women had received Tdap vaccination) [38]. However, this information was not available for the whole study population. Epidural anesthesia is often associated with prolonged labor and maternal fever [63, 64], often leading to prophylactic antibiotic use [64]. Transient or non-specific maternal fever might thus get coded as chorioamnionitis [62], leading to an overcoding of this diagnosis in women who had received epidural anesthesia.

As vaccinated women in the 3 studies with a significant association between Tdap-vaccination and chorioamnionitis obtained better prenatal care (earlier and more frequent ante-natal clinic visits [38] and more frequently ultrasound examinations [40]), they may also have requested epidural anesthesia more frequently than non-vaccinated women. Unfortunately, rates of epidural anesthesia were not analyzed in any of the studies investigating chorioamnionitis after Tdap vaccination.

In our systematic review, vaccine effectiveness data of 855,546 mother-infant-pairs from Australia, Spain, UK, and the USA were analyzed. In the USA, Tdap vaccination in pregnancy has been recommended since 2011 [65], in Australia and Valencia (Spain) since 2015 [46, 48]. In the UK, pertussis vaccination in pregnancy was introduced in 2012 as an emergency measure during a nationwide pertussis outbreak with 14 infant deaths [13]. In 2014, it was decided to continue with the program, since pertussis incidence remained high in the overall population and the available evidence showed good safety and effectiveness of the intervention [16, 66]. Compared to safety studies, VE studies in our review had a lower risk of bias and higher quality of evidence. In all studies considering laboratory confirmed pertussis as the outcome, VE was high: it ranged from 69 to 91% for prevention of pertussis, from 91 to 94% for prevention of hospitalization and was 95% for prevention of death in infants 0–3 months of age. The effect was diluted by additional inclusion of clinically suspected cases without laboratory pertussis confirmation in the study by Becker-Dreps [45], resulting in lower vaccine effectiveness estimates.

Our up-to-date systematic review has several strengths. We focus entirely on clinical outcomes (rather than immunological [serological] markers) and include a critical evaluation of a recently detected possible safety signal, i.e. chorioamnionitis. Using ROBINS-I, we applied the most advanced ROB tool to assess internal validity of the included observational studies, allowing a very detailed judgement. However, our review also has limitations that are mainly due to the limitations of the included studies. The majority of studies investigating safety outcomes had a considerable risk of bias, which impairs the ability of drawing firm conclusions on the risk of adverse events. Moreover, the three RCTs were designed and powered for the assessment of the immune response in pregnancy and, thus, were hampered by participant numbers that were too small for the assessment of rare safety outcomes. Regarding studies that investigated VE outcomes, those with the highest numbers of participants used the screening method to calculate VE. Since this method uses population estimates rather than individual data, controlling for confounders was not possible.

## Conclusions

In this systematic review we summarize the currently available evidence on safety and effectiveness of pertussis vaccination in pregnancy. Vaccine effectiveness for prevention of infant pertussis, hospitalization and death is high. Two safety issues were observed in the included studies: fever and chorioamnionitis. Six additional cases of fever per 100,000 vaccinated women are to be expected, which is a small number and makes fever an adverse event of minor importance. Increased ICD-coding of chorioamnionitis, even though statistically associated with Tdap vaccination during pregnancy in some of the studies, does not seem to be clinically relevant. However, when implementing Tdap vaccination in pregnancy, surveillance of all safety endpoints, including chorioamnionitis and its sequelae, is needed in view of a likely residual healthy vaccinee bias in currently available studies and in view of the overall low quality of the evidence. Given the high vaccine effectiveness, pertussis vaccination during pregnancy has an overall positive benefit-risk ratio, particularly if the incidence of pertussis in infancy is high.

## Supplementary information

**Additional file 1: Figure S1.** Systematic review on safety and effectiveness of pertussis vaccination in pregnancy; flowchart selection of included studies. **Table S1.** Systematic review on safety and effectiveness of pertussis vaccination in pregnancy; excluded studies. **Table S2.** Systematic review on safety and effectiveness of pertussis vaccination in pregnancy; results of the risk of bias (ROB) assessment. **Table S3.** Systematic review on safety and effectiveness of pertussis vaccination in pregnancy; GRADE Evidence profile.

## Data Availability

The full dataset is available from the corresponding author upon request.

## Abbreviations

95% CI: 95% confidence interval
GRADE: Grading of Recommendations Assessment, Development and Evaluation
ICD: International classification of diseases
LBW: Low birth weight
NICU: Neonatal intensive care unit
PICO: Population, intervention, comparator, outcome
PRISMA: Preferred Reporting Items for Systematic Reviews and Meta-analyses
RCT: Randomized controlled trial
RoB: Risk of bias
RR: Relative risk
Tdap: Tetanus-diphtheria-acellular pertussis
VE: Vaccine effectiveness
VLBW: Very low birth weight
