# Supplementary material for: Safety and effectiveness of acellular pertussis vaccination during pregnancy: a systematic review
Source: BMC Infect Dis. 2020 Feb 13;20:136. doi: 10.1186/s12879-020-4824-3 (PMC7020352; doi:10.1186/s12879-020-4824-3)
Supplement: Supplementary file 1 — Additional file 1: Figure S1. Systematic review on safety and effectiveness of pertussis vaccination in pregnancy; flowchart selection of included studies. Table S1. Systematic review on safety and effectiveness of pertussis vaccination in pregnancy; excluded studies. Table S2. Systematic review on safety and effectiveness of pertussis vaccination in pregnancy; results of the risk of bias (ROB) assessment. Table S3. Systematic review on safety and effectiveness of pertussis vaccination in pregnancy; GRADE Evidence profile. [file 12879_2020_4824_MOESM1_ESM.pdf]

Safety and effectiveness of acellular pertussis vaccination during pregnancy: a systematic review

Additional file 1:

Figure S1: Systematic review on safety and effectiveness of pertussis vaccination in pregnancy; flowchart selection of included studies

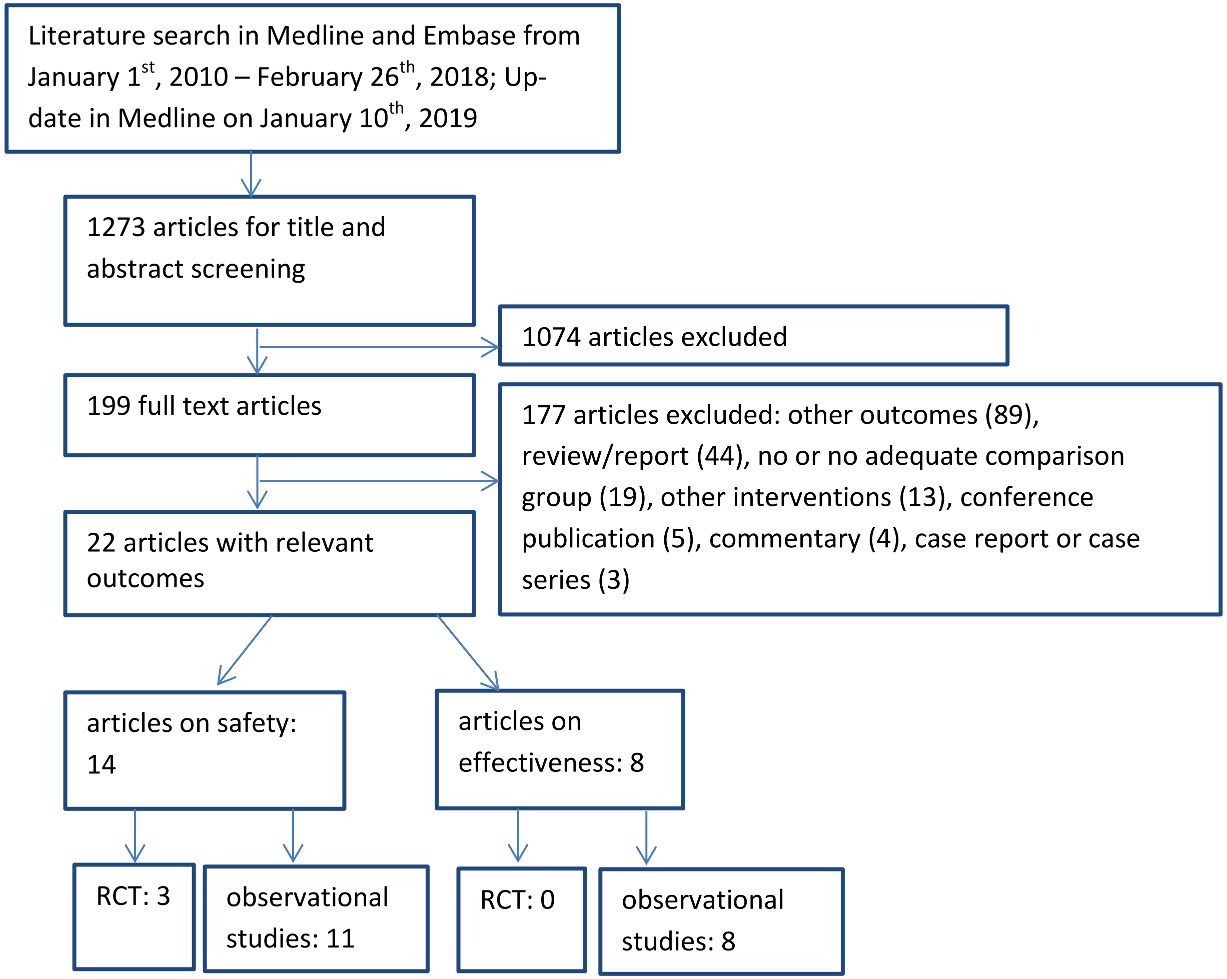

Table S1: Systematic review on safety and effectiveness of pertussis vaccination in pregnancy; excluded studies

**Reason for exclusion: other outcomes (n=89)**
Abraham, C., Pichichero, M., Eisenberg, J., & Singh, S. (2018). Third-trimester maternal vaccination against pertussis and pertussis antibody concentrations. *Obstetrics & Gynecology*, *131*(2), 364-369.
Abu Raya B, Bamberger E, Almog M, Peri R, Srugo I, Kessel A. Immunization of pregnant women against pertussis: The effect of timing on antibody avidity. Vaccine. 2015;33(16):1948-52.
Abu Raya B, Srugo I, Kessel A, Peterman M, Bader D, Gonen R, et al. The effect of timing of maternal tetanus, diphtheria, and acellular pertussis (Tdap) immunization during pregnancy on newborn pertussis antibody levels – A prospective study. Vaccine. 2014;32(44):5787-93.
Abu Raya, B., Srugo, I., & Bamberger, E. (2016). Optimal timing of immunization against pertussis during pregnancy. Clinical Infectious Diseases, 63(1), 143-144.
Abu Raya, B., Srugo, I., Kessel, A., Peterman, M., Vaknin, A., & Bamberger, E. (2015). The decline of pertussis-specific antibodies after tetanus, diphtheria, and acellular pertussis immunization in late pregnancy. *The Journal of infectious diseases*, *212*(12), 1869-1873.
Atkins, K. E., Fitzpatrick, M. C., Galvani, A. P., & Townsend, J. P. (2016). Cost-effectiveness of pertussis vaccination during pregnancy in the United States. American journal of epidemiology, 183(12), 1159-1170.
Becerra-Culqui TA(1), Getahun D(2), Chiu V(2), Sy LS(2), Tseng HF. Prenatal Tetanus, Diphtheria, Acellular Pertussis Vaccination and Autism Spectrum Disorder. Pediatrics. 2018 Sep;142(3). pii: e20180120. doi: 10.1542 /peds.2018-0120. Epub2018 Aug 13
Bento, A. I., & Rohani, P. (2016). Forecasting epidemiological consequences of maternal immunization. Clinical Infectious Diseases, 63(suppl_4), S205-S212.
Bosch, J. J., Teper, A., Pollak, F., Libster, R., & Rivarola, M. R. (2016). seroprevalence Of Bordetella Pertussis In Pregnant Women And Newborns: 016. Pediatric Pulmonology, 51, S11.
Bosch, J., Fernández, H., Polak, F., Musante, G., Libster, R., & Rocca, M. (2017). Seroprevalence of Bordetella pertussis among vaccinated and unvaccinated pregnant women and newborn infants in a university hospital of Buenos Aires. Arch Argent Pediatr, 115(4), 311-315.
Byrne, L., Campbell, H., Andrews, N., Ribeiro, S., & Amirthalingam, G. (2018). Hospitalisation of preterm infants with pertussis in the context of a maternal vaccination programme in England. Archives of disease in childhood, 103(3), 224-229.
Caboré RN, Maertens K, Dobly A, Leuridan E, Van Damme P, K. H. Influence of maternal vaccination against diphtheria, tetanus, and pertussis on the avidity of infant antibody responses to a pertussis containing vaccine in Belgium. Virulence. 2017;8(7):1245-54.
Choi, Y. H., Campbell, H., Amirthalingam, G., Van Hoek, A. J., & Miller, E. (2016). Investigating the pertussis resurgence in England and Wales, and options for future control. BMC medicine, 14(1), 121.
Donaldson, B., Jain, P., Holder, B. S., Lindsey, B., Regan, L., & Kampmann, B. (2015). What determines uptake of pertussis vaccine in pregnancy? A cross sectional survey in an ethnically diverse population of pregnant women in London. Vaccine, 33(43), 5822-5828.
Eberhardt, C. S., Blanchard-Rohner, G., Lemaître, B., Boukrid, M., Combescure, C., Othenin-Girard, V.,& Siegrist, C. A. (2016). Maternal immunization earlier in pregnancy maximizes antibody transfer and expected infant seropositivity against pertussis. Clinical Infectious Diseases, 62(7), 829-836.
Eberhardt, C. S., Blanchard-Rohner, G., Lemaître, B., Combescure, C., Othenin-Girard, V., Chilin, A. & Siegrist, C. A. (2017). Pertussis antibody transfer to preterm neonates after second-versus third-trimester maternal immunization. Clinical Infectious Diseases, 64(8), 1129-1132.
Ercan, T. E., Sonmez, C., Vural, M., Erginoz, E., Torunoğlu, M. A., & Perk, Y. (2013). Seroprevalance of pertussis antibodies in maternal and cord blood of preterm and term infants. *Vaccine*, *31*(38), 4172-4176.
Erratum: Antibody Responses After Primary Immunization in Infants Born to Women Receiving a Pertussis-containing Vaccine During Pregnancy: Single Arm Observational Study With a Historical Comparator (Clinical Infectious Diseases (2015) 61 (1637-44)
Fallo, A. A., Neyro, S. E., Manonelles, G. V., Lara, C., Hozbor, D., Zintgraff, J.& Canle, O. (2016). Prevalence of pertussis antibodies in maternal blood, cord serum, and infants from mothers with and those without Tdap booster vaccination during pregnancy in Argentina. Journal of the Pediatric Infectious Diseases Society, 7(1), 11-17.
Fernández-Cano, M. I., Gil, L. A., & Martí, M. C. (2015). Cost–benefit of the introduction of new strategies for vaccination against pertussis in Spain: Cocooning and pregnant vaccination strategies. Vaccine, 33(19), 2213-2220.

Feunou, P. F., Mielcarek, N., & Locht, C. (2016). Reciprocal interference of maternal and infant immunization in protection against pertussis. Vaccine, 34(8), 1062-1069.
Gaillard, M. E., Bottero, D., Zurita, M. E., Carriquiriborde, F., Martin Aispuro, P., Bartel, E.,& Hozbor, D. F. (2017). Pertussis Maternal immunization: narrowing the Knowledge gaps on the Duration of Transferred Protective immunity and on Vaccination Frequency. Frontiers in immunology, 8, 1099.
Gall, S. A., Myers, J., & Pichichero, M. (2011). Maternal immunization with tetanus–diphtheria–pertussis vaccine: effect on maternal and neonatal serum antibody levels. American journal of obstetrics and gynecology, 204(4), 334-e1.
Gentile, A., del Valle Juarez, M., Lucion, M. F., Martínez, A. C., Romanin, V., Areso, S., & Mistchenko, A. (2018). Bordetella pertussis (Bp) disease: Before (2003–2011) and after (2013–2016) maternal immunization strategy in a pediatric hospital. *Vaccine*, *36*(11), 1375-1380.
Gesser-Edelsburg, A., Shir-Raz, Y., Hayek, S., Aassaraf, S., & Lowenstein, L. (2017). Despite awareness of recommendations, why do health care workers not immunize pregnant women? American journal of infection control, 45(4), 436-439.
Goldfarb, I., Jennewein, M., Cosgrove, C., Brown, J., Krykbaeva, M., Cooperrider, J. & Riley, L. (2017). Maternal Tdap: how do antibodies protect newborns against pertussis? American Journal of Obstetrics and Gynecology, 216(1).
Gray, L., Quataert, S., Secor-Socha, S., Thornburg, L., Grzesik, K., Muthukrishnan, G., & Mossman, T. (2016, March). Antibody Responses to Pertussis Vaccination in Pregnant Versus Non-Pregnant Subjects. In REPRODUCTIVE SCIENCES (Vol. 23, pp. 82A-82A). 2455 TELLER RD, THOUSAND OAKS, CA 91320 USA: SAGE PUBLICATIONS INC.
Grizas, A. P., Camenga, D., & Vázquez, M. (2012). Cocooning: a concept to protect young children from infectious diseases. Current opinion in pediatrics, 24(1), 92-97.
Hardy-Fairbanks, A. J., Pan, S. J., Decker, M. D., Johnson, D. R., Greenberg, D. P., Kirkland, K. B. & Bernstein, H. H. (2013). Immune responses in infants whose mothers received Tdap vaccine during pregnancy. The Pediatric infectious disease journal, 32(11), 1257-1260.
Healy C, Rench MA, Swaim LS, et al. Association between third-trimester tdap immunization and neonatal pertussis antibody concentration. JAMA. 2018;320(14):1464-70.
Healy, C. M., Rench, M. A., & Baker, C. J. (2012). Importance of timing of maternal combined tetanus, diphtheria, and acellular pertussis (Tdap) immunization and protection of young infants. Clinical infectious diseases, 56(4), 539-544.
Healy, C. M., Rench, M. A., Wootton, S. H., & Castagnini, L. A. (2015). Evaluation of the impact of a pertussis cocooning program on infant pertussis infection. The Pediatric infectious disease journal, 34(1), 22-26.
Heininger, U., Riffelmann, M., Bär, G., Rudin, C., & von König, C. H. W. (2013). The protective role of maternally derived antibodies against Bordetella pertussis in young infants. The Pediatric infectious disease journal, 32(6), 695-698.
Hincapié-Palacio D(1), Hoyos MC(2), Ochoa J(2), Montoya N(2), García D(3), Osorio E(3); Pertussis Working Group. Effect of maternal immunization against pertussis in Medellin and the metropolitan area, Colombia, 2016-2017. Vaccine. 2018 Jun 22;36(27):3984-3991. doi: 10.1016/j.vaccine.2018.05.020. Epub2018 May 19.
Huygen, K., Caboré, R. N., Maertens, K., Van Damme, P., & Leuridan, E. (2015). Humoral and cell mediated immune responses to a pertussis containing vaccine in pregnant and nonpregnant women. Vaccine, 33(33), 4117-4123.
Huynh, J., & McMullan, B. (2016). Antibody responses in infants after antenatal maternal pertussis vaccination. Journal of paediatrics and child health, 52(8), 850-850.
Jones, C., Pollock, L., Barnett, S. M., Battersby, A., & Kampmann, B. (2013). Specific antibodies against vaccine-preventable infections: a mother–infant cohort study. BMJ open, 3(4), e002473.
Jones, C., Pollock, L., Barnett, S. M., Battersby, A., & Kampmann, B. (2014). The relationship between concentration of specific antibody at birth and subsequent response to primary immunization. Vaccine, 32(8), 996-1002.
Kara EO, Campbell H, Ribeiro S, Fry NK, Litt D, Eletu S, et al. Survey of household contacts of infants with laboratory-confirmed pertussis infection during a national pertussis outbreak in England and Wales. Pediatr Infect Dis J. 2017;36(2):140-5.
Kent, A., Ladhani, S. N., Andrews, N. J., Matheson, M., England, A., Miller, E. & PUNS study group. (2016). Pertussis Antibody Concentrations in Infants Born Prematurely to Mothers Vaccinated in Pregnancy. Pediatrics, 138(1).
Kharbanda, E. O., Vazquez-Benitez, G., Lipkind, H., Naleway, A. L., Klein, N. P., Cheetham, T. C., & Nordin, J. D. (2014). Receipt of pertussis vaccine during pregnancy across 7 Vaccine Safety Datalink sites. Preventive medicine, 67, 316-319.
Khodr, Z. G., Bukowinski, A. T., Gumbs, G. R., & Conlin, A. M. S. (2017). Tetanus, diphtheria, and acellular pertussis vaccination during pregnancy and reduced risk of infant acute respiratory infections. Vaccine, 35(42), 5603-5610.
Ladhani, S. N., Andrews, N. J., Southern, J., Jones, C. E., Amirthalingam, G., Waight, P. A.& Burbidge, P. (2015). Antibody responses after primary immunization in infants born to women receiving

a pertussis-containing vaccine during pregnancy: single arm observational study with a historical comparator. Clinical infectious diseases, 61(11), 1637-1644.
Leuridan, E., Hens, N., Peeters, N., de Witte, L., Van der Meeren, O., & Van Damme, P. (2011). Effect of a prepregnancy pertussis booster dose on maternal antibody titers in young infants. The Pediatric infectious disease journal, 30(7), 608-610.
Lugnér, A. K., van der Maas, N., van Boven, M., Mooi, F. R., & de Melker, H. E. (2013). Cost-effectiveness of targeted vaccination to protect new-borns against pertussis: comparing neonatal, maternal, and cocooning vaccination strategies. Vaccine, 31(46), 5392-5397.
MacDougall, D. M., Halperin, B. A., Langley, J. M., McNeil, S. A., MacKinnon-Cameron, D., Li, L., & Halperin, S. A. (2016). Knowledge, attitudes, beliefs, and behaviors of pregnant women approached to participate in a Tdap maternal immunization randomized, controlled trial. Human vaccines & immunotherapeutics, 12(4), 879-885.
Maertens K, Caboré RN, Huygen K, Vermeiren S, Hens N, Van Damme P, et al. Pertussis vaccination during pregnancy in Belgium: Follow-up of infants until 1 month after the fourth infant pertussis vaccination at 15 months of age. 2016;34(31):3613-9.
Maertens, K., Braeckman, T., Top, G., Van Damme, P., & Leuridan, E. (2016). Maternal pertussis and influenza immunization coverage and attitude of health care workers towards these recommendations in Flanders, Belgium. *Vaccine*, *34*(47), 5785-5791.
Maertens, K., Burbidge, P., Van Damme, P., Goldblatt, D., & Leuridan, E. (2017). Pneumococcal immune response in infants whose mothers received tetanus, diphtheria and acellular pertussis vaccination during pregnancy. The Pediatric infectious disease journal, 36(12), 1186-1192.
Maertens, K., Caboré, R. N., Huygen, K., Vermeiren, S., Hens, N., Van Damme, P., & Leuridan, E. (2016). Pertussis vaccination during pregnancy in Belgium: follow-up of infants until 1 month after the fourth infant pertussis vaccination at 15 months of age. Vaccine, 34(31), 3613-3619.
Maertens, K., Hoang, T. T. H., Nguyen, T. D., Caboré, R. N., Duong, T. H., Huygen, K. & Leuridan, E. (2016). The effect of maternal pertussis immunization on infant vaccine responses to a booster pertussis-containing vaccine in Vietnam. Clinical Infectious Diseases, 63(suppl_4), S197-S204.
Maertens, K., Hoang, T. T. H., Nguyen, T. D., Caboré, R. N., Duong, T. H., Huygen, K., Hens, N., Van Damme, P., Dang, D. A. and Leuridan, E.
Maertens, K., Tran, T. M. P., Hens, N., Van Damme, P., & Leuridan, E. (2017). Effect of prepregnancy pertussis vaccination in young infants. The Journal of infectious diseases, 215(12), 1855-1861.
McAuslane, H., Andrews, N., Coole, L., & Wensley, A. (2014). A survey of maternal pertussis vaccine uptake in England. International Journal of Infectious Diseases, 21, 434.
McHugh L(1), Marshall HS(2), Perrett KP(3), Nolan T(3), Wood N(4), Lambert SB(5),Richmond P(6), Ware RS(7), Binks P(1), Binks MJ(1), Andrews RM(1)(8). The Safety of Influenza and Pertussis Vaccination in Pregnancy in a Cohort of Australian Mother-Infant Pairs, 2012-2015: The FluMum Study. Clin Infect Dis. 2018 Nov 23. doi: 10.1093/cid/ciy517
Naidu, M. A., Muljadi, R., Davies-Tuck, M. L., Wallace, E. M., & Giles, M. L. (2016). The optimal gestation for pertussis vaccination during pregnancy: a prospective cohort study. American journal of obstetrics and gynecology, 215(2), 237-e1.
Naidu, M., Wallace, E., & Giles, M. (2015, April). Pertussis vaccine: when is the optimal time to vaccinate during pregnancy?. In BJOG-AN INTERNATIONAL JOURNAL OF OBSTETRICS AND GYNAECOLOGY (Vol. 122, pp. 365-365). 111 RIVER ST, HOBOKEN 07030-5774, NJ USA: WILEY-BLACKWELL.
Perry, J., Towers, C. V., Weitz, B., & Wolfe, L. (2017). Patient reaction to Tdap vaccination in pregnancy. Vaccine, 35(23), 3064-3066.
Pesco, P., Bergero, P., Fabricius, G., & Hozbor, D. (2014). Modelling the effect of changes in vaccine effectiveness and transmission contact rates on pertussis epidemiology. Epidemics, 7, 13-21.
Plans, P., Álvarez, E., De Ory, F., Campins, M., Payà, T., Balfagón, P. & Domínguez, A. (2014). Prevalence of antibody to Bordetella pertussis in neonates and prevalence of recent pertussis infection in pregnant women in Catalonia (Spain) in 2003 and 2013. The Pediatric infectious disease journal, 33(11), 1114-1118.
Poirrier, J. E., Mungall, B., Lee, I. H., Terlinden, A., & Curran, D. (2014). Cost-effectiveness of maternal immunisation for pertussis in New Zealand. Value in Health, 17(7), A806.
Quinello, C., Quintilio, W., Carneiro-Sampaio, M., & Palmeira, P. (2010). Passive Acquisition of Protective Antibodies Reactive with Bordetella pertussis in Newborns via Placental Transfer and Breast-feeding. Scandinavian journal of immunology, 72(1), 66-73.
Raya, B. A., Bamberger, E., Almog, M., Peri, R., Srugo, I., & Kessel, A. (2015). Immunization of pregnant women against pertussis: the effect of timing on antibody avidity. *Vaccine*, *33*(16), 1948-1952.
Raya, B. A., Srugo, I., Kessel, A., Peterman, M., Bader, D., Gonen, R., & Bamberger, E. (2014). The effect of timing of maternal tetanus, diphtheria, and acellular pertussis (Tdap) immunization during pregnancy on newborn pertussis antibody levels–a prospective study. Vaccine, 32(44), 5787-5793.
Raya, B. A., Srugo, I., Kessel, A., Peterman, M., Bader, D., Peri, R.& Bamberger, E. (2014). The induction of breast milk pertussis specific antibodies following gestational tetanus–diphtheria–acellular pertussis vaccination. *Vaccine*, *32*(43), 5632-5637.
Saffar, M. J., Ajami, A., Moslemizadeh, N., Khalilian, A., Saffar, H., & Shojai, J. (2013). Pertussis Seroimmunity and One Year Immunogenicity of Combined Adult Type Pertussis Vaccine in Pre

marriage Girls, 2009-2011. Journal of Mazandaran University of Medical Sciences, 23(98), 190-198.
Sartori AMC, de Soárez PC, Fernandes EG, Gryninger LCF, Viscondi JYK, Novaes HMD. Cost-effectiveness analysis of universal maternal immunization with tetanus-diphtheria-acellular pertussis (Tdap) vaccine in Brazil. Vaccine. 2016;34(13):1531-9.
Shakib JH, Ralston S, Raissy HH, Stoddard GJ, Edwards KM, Byington CL. Pertussis antibodies in postpartum women and their newborns. J Perinatol. 2010;30(2):93-7.
Smallenburg LCS, van Welie NA, Elvers LH, van Huisseling JCM, Teunis PFM, Versteegh FGA. Decline of IgG pertussis toxin measured in umbilical cord blood, and neonatal and early infant serum. Eur J Clin Microbiol Infect Dis. 2014;33(9):1541-5.
Smallenburg, L. C. S., Van Welie, N. A., Elvers, L. H., van Huisseling, J. C. M., Teunis, P. F. M., & Versteegh, F. G. A. (2014). Decline of IgG pertussis toxin measured in umbilical cord blood, and neonatal and early infant serum. *European journal of clinical microbiology & infectious diseases*, *33*(9), 1541-1545.
Sukumaran L, McCarthy NL, Kharbanda EO, et al. Association of tdap vaccination with acute events and adverse birth outcomes among pregnant women with prior tetanus-containing immunizations. JAMA. 2015;314(15):1581-7.
Sukumaran, L., McCarthy, N. L., Kharbanda, E. O., McNeil, M. M., Naleway, A. L., Klein, N. P., ... & Weintraub, E. S. (2015). Association of Tdap vaccination with acute events and adverse birth outcomes among pregnant women with prior tetanus-containing immunizations. *Jama*, *314*(15), 1581-1587.
Sukumaran, L., McCarthy, N. L., Kharbanda, E. O., Vazquez-Benitez, G., Lipkind, H. S., Jackson, L., ... & Kawai, A. T. (2018). Infant hospitalizations and mortality after maternal vaccination. *Pediatrics*, *141*(3), e20173310.
Sukumaran, L., McCarthy, N. L., Kharbanda, E. O., Weintraub, E., Vazquez-Benitez, G., McNeil, M. M. & Lugg, M. M. (2015). Safety of tetanus, diphtheria, and acellular pertussis and influenza vaccinations in pregnancy. *Obstetrics and gynecology*, *126*(5), 1069.
Terranella, A., Asay, G. R. B., Messonnier, M. L., Clark, T. A., & Liang, J. L. (2013). Pregnancy dose Tdap and postpartum cocooning to prevent infant pertussis: a decision analysis. Obstetrical & Gynecological Survey, 68(9), 615-616.
Türkoğlu, E., Sönmez, C., Özer, E., Çöplü, N., & Kurugöl, Z. (2016). Low pertussis antibody levels in maternal and umbilical cord blood samples in Turkey. The Turkish journal of pediatrics, 58(6), 573-578.
van den Berg, J. P., Westerbeek, E. A., Berbers, G. A., van Gageldonk, P. G., van der Klis, F. R., & van Elburg, R. M. (2010). Transplacental transport of IgG antibodies specific for pertussis, diphtheria, tetanus, haemophilus influenzae type b, and Neisseria meningitidis serogroup C is lower in preterm compared with term infants. The Pediatric infectious disease journal, 29(9), 801-805.
van Hoek, A. J., Andrews, N., Campbell, H., Amirthalingam, G., Edmunds, W. J., & Miller, E. (2013). The social life of infants in the context of infectious disease transmission; social contacts and mixing patterns of the very young. PloS one, 8(10), e76180.
van Hoek, A. J., Campbell, H., Amirthalingam, G., Andrews, N., & Miller, E. (2016). Cost-effectiveness and programmatic benefits of maternal vaccination against pertussis in England. Journal of Infection, 73(1), 28-37.
Vilajeliu, A., Ferrer, L., Munrós, J., Goncé, A., López, M., Costa, J., ... & PERTU Working Group. (2016). Pertussis vaccination during pregnancy: antibody persistence in infants. Vaccine, 34(33), 3719-3722.
Villarreal Perez, J. Z., Ramírez Aranda, J. M., de la O Cavazos, M., Zamudio Osuna, M. D. J., Perales Dávila, J., Ballesteros Elizondo, M. R. & Rodríguez González, A. M. (2017). Randomized clinical trial of the safety and immunogenicity of the Tdap vaccine in pregnant Mexican women. *Human vaccines & immunotherapeutics*, *13*(1), 128-135.
Villarreal-Pérez, J. Z., Ramírez-Aranda, J. M., Rodríguez-Rodríguez, I., Perales-Dávila, J., García-Elizondo, F. J., Gómez-Gómez, C., ... & Ballesteros-Elizondo, R. (2014). Absence of antibodies against Bordetella pertussis in pregnant women and newborns in the state of Nuevo Leon. Journal of perinatal medicine, 42(5), 649-654.
Voysey, M., Kelly, D. F., Fanshawe, T. R., Sadarangani, M., O'brien, K. L., Perera, R., & Pollard, A. J. (2017). The influence of maternally derived antibody and infant age at vaccination on infant vaccine responses: an individual participant meta-analysis. JAMA pediatrics, 171(7), 637-646.
Wanlapakorn, N., Thongmee, T., Vichaiwattana, P., Leuridan, E., Vongpunsawad, S., & Poovorawan, Y. (2017). Antibodies to Bordetella pertussis antigens in maternal and cord blood pairs: a Thai cohort study. PeerJ, 5, e4043.
Warfel, J. M., Papin, J. F., Wolf, R. F., Zimmerman, L. I., & Merkel, T. J. (2014). Maternal and neonatal vaccination protects newborn baboons from pertussis infection. The Journal of infectious diseases, 210(4), 604-610.
Westra, T. A., de Vries, R., Tamminga, J. J., Sauboin, C. J., & Postma, M. J. (2010). Cost-effectiveness analysis of various pertussis vaccination strategies primarily aimed at protecting infants in the

Netherlands. Clinical therapeutics, 32(8), 1479-1495.
Winter, K., Cherry, J. D., & Harriman, K. (2016). Effectiveness of prenatal tetanus, diphtheria, and acellular pertussis vaccination on pertussis severity in infants. Clinical Infectious Diseases, ciw633.
Zerbo, O., Chan, B., Goddard, K., Lewis, N., Bok, K., Klein, N. P., & Baxter, R. (2016). Kaiser Permanente Northern California pregnancy database: Description and proof of concept study. Vaccine, 34(46), 5519-5523.
Zhu, Y., van Boemmel-Wegmann, S., & Albogami, Y. (2016). Repeat Tdap Vaccination and Adverse Birth Outcomes. Jama, 315(12), 1285-1286.
**Reason for exclusion: review/report (n=44)**
Abu Raya B, Edwards KM, Scheifele DW, Halperin SA. Pertussis and influenza immunisation during pregnancy: a landscape review. Lancet Infect Dis. 2017.
Amirthalingam G, Letley L, Campbell H, Green D, Yarwood J, Ramsay M. Lessons learnt from the implementation of maternal immunization programs in England. Hum Vaccin Immunother. 2016;12(11):2934-9.
Amirthalingam, G. (2013). Strategies to control pertussis in infants. Archives of disease in childhood, 98(7), 552-555.
Amirthalingam, G., Letley, L., Campbell, H., Green, D., Yarwood, J., & Ramsay, M. (2016). Lessons learnt from the implementation of maternal immunization programs in England. Human vaccines & immunotherapeutics, 12(11), 2934-2939.
Augustynowicz, E., Lutyńska, A., Piotrowska, A., & Paradowska-Stankiewicz, I. (2017). The safety and effectiveness of vaccination against influenza and pertussis in pregnant women. Przeglad epidemiologiczny, 71(1), 55-67.
Baclic O, Tunis M, Rotondo J, Saboui M, Duchesne-Belanger S, Brophy J, et al. Literature Review on Immunization in Pregnancy with Tetanus Toxoid, Reduced Diphtheria Toxoid and Reduced Acellular Pertussis (Tdap) Vaccine: Safety, Immunogenicity and Effectiveness: An Advisory Committee Review Advisory Committee on Immunization (NACI). Ottawa: National Advisory Committee on Immunization (NACI); 2018.
Bento, A. I., King, A. A., & Rohani, P. (2017). Maternal pertussis immunisation: clinical gains and epidemiological legacy. Eurosurveillance, 22(15).
Bethancourt, C. N., Wang, T. L., & Bocchini, J. A. (2017). Vaccination during pregnancy: First line of defense for expecting mothers and vulnerable young infants. Current opinion in pediatrics, 29(6), 737-743.
Blanchard-Rohner, G., & Eberhardt, C. (2017). Review of maternal immunisation during pregnancy: focus on pertussis and influenza. Swiss medical weekly, 147(4344).
Bont, L. J., Bekker, M. N., & Rots, N. (2016). Infection prevention in newborns through maternal vaccination: current insights and developments. Nederlands tijdschrift voor geneeskunde, 160, D411-D411.
Campbell H, Gupta S, Dolan GP, Kapadia SJ, Kumar Singh A, Andrews N, et al. Review of vaccination in pregnancy to prevent pertussis in early infancy. J Med Microbiol. 2018;67(10):1426-56.
Campbell, P. T., McVernon, J., & Geard, N. (2017). Determining the best strategies for maternally targeted pertussis vaccination using an individual-based model. American journal of epidemiology, 186(1), 109-117.
Cherry JD. Pertussis in Young Infants Throughout the World. Clin Infect Dis. 2016;63(suppl_4):S119-S22.
Eberhardt, C. S., & Siegrist, C. A. (2017). What is wrong with pertussis vaccine immunity? Inducing and recalling vaccine-specific immunity. Cold Spring Harbor perspectives in biology, 9(12), a029629.
Furuta M, Sin J, Ng ESW, Wang K. Efficacy and safety of pertussis vaccination for pregnant women - a systematic review of randomised controlled trials and observational studies. BMC pregnancy and childbirth. 2017;17(1):390.
Gkentzi D, Katsakiori P, Marangos M, Hsia Y, Amirthalingam G, Heath PT, et al. Maternal vaccination against pertussis: a systematic review of the recent literature. Arch Dis Child Fetal Neonatal Ed. 2017.
Hartzell JD, Blaylock JM. Whooping cough in 2014 and beyond: An update and review. Chest. 2014;146(1):205-14.
Keller-Stanislawski, B., Englund, J. A., Kang, G., Mangtani, P., Neuzil, K., Nohynek, H., ... & Zuber, P. (2014). Safety of immunization during pregnancy: a review of the evidence of selected inactivated and live attenuated vaccines. Vaccine, 32(52), 7057-7064.
Kilgore PE, Salim AM, Zervos MJ, Schmitt H-J. Pertussis: Microbiology, Disease, Treatment, and Prevention. Clin Microbiol Rev. 2016;29(3):449-86.
Kuehn, B. M. (2010). Panel backs wider pertussis vaccination to curb outbreaks, prevent deaths. JAMA, 304(24), 2684-2686.

Leuridan, E. (2017). Pertussis vaccination in pregnancy: state of the art. vaccine, 35(35), 4453-4456.
Leuridan, E., Hoang, T. T. H., Dang, D. A. and Van Damme, P. Pertussis Vaccination and Pregnancy. Clinical Infectious Diseases, Volume 57, Issue 3, 1 August 2013, Pages 471–472, https://doi.org/10.1093/cid/cit217
Maertens, K., De Schutter, S., Braeckman, T., Baerts, L., Van Damme, P., De Meester, I., & Leuridan, E. (2014). Breastfeeding after maternal immunisation during pregnancy: providing immunological protection to the newborn: a review. Vaccine, 32(16), 1786-1792.
McMillan M, Clarke M, Parrella A, Fell DB, Amirthalingam G, Marshall HS. Safety of Tetanus, Diphtheria, and Pertussis Vaccination During Pregnancy: A Systematic Review. Obstet Gynecol. 2017;129(3):560-73.
Niewiesk S. Maternal Antibodies: Clinical Significance, Mechanism of Interference with Immune Responses, and Possible Vaccination Strategies. Frontiers in immunology. 2014;5:446.
Omer, S. B. (2017). Maternal immunization. New England Journal of Medicine, 376(13), 1256-1267.
Perrett, K. P., & Nolan, T. M. (2017). Immunization during pregnancy: impact on the infant. Pediatric Drugs, 19(4), 313-324.
Raya, B. A., Edwards, K. M., Scheifele, D. W., & Halperin, S. A. (2017). Pertussis and influenza immunisation during pregnancy: a landscape review. *The Lancet Infectious Diseases*, *17*(7), e209-e222.
Swamy, G. K., & Beigi, R. H. (2015). Maternal benefits of immunization during pregnancy. Vaccine, 33(47), 6436-6440.
Swamy, G. K., & Garcia-Putnam, R. (2013). Vaccine-preventable diseases in pregnancy. American journal of perinatology, 30(02), 089-098.
Swamy, G. K., & Garcia-Putnam, R. (2014). Maternal immunization to benefit the mother, fetus, and infant. Obstetrics and Gynecology Clinics, 41(4), 521-534.
Swamy, G. K., & Heine, R. P. (2015). Vaccinations for pregnant women. Obstetrics and gynecology, 125(1), 212.
Switzer, C., Dukanovic, J., Weibel, D., & Sturkenboom, M. (2017, August). Identifying GAIA Outcomes in Maternal Immunization Studies. In PHARMACOEPIDEMIOLOGY AND DRUG SAFETY (Vol. 26, pp. 619-619). 111 RIVER ST, HOBOKEN 07030-5774, NJ USA: WILEY.
Urwyler, P., & Heininger, U. (2014). Protecting newborns from pertussis–the challenge of complete cocooning. BMC infectious diseases, 14(1), 397.
van den Biggelaar, A. H., & Poolman, J. T. (2016). Predicting future trends in the burden of pertussis in the 21st century: implications for infant pertussis and the success of maternal immunization. Expert review of vaccines, 15(1), 69-80.
Verweij, M., Lambach, P., Ortiz, J. R., & Reis, A. (2016). Maternal immunisation: ethical issues. The Lancet Infectious Diseases, 16(12), e310-e314.
Vilajeliu, A., García-Basteiro, A. L., & Bayas, J. M. (2015). Protecting newborns against pertussis: the value of vaccinating during pregnancy.
Vilca, L. M., & Esposito, S. (2018). The crucial role of maternal care providers as vaccinators for pregnant women. Vaccine, 36(36), 5379-5384.
Villena, R., Vidal, P., Carrillo, F., & Salinas, M. (2017). Pertussis vaccination in pregnancy: security and effectiveness in the protection of the infant. Revista chilena de pediatria, 88(3), 318-323.
Wiley, K. E., Zuo, Y., Macartney, K. K., & McIntyre, P. B. (2013). Sources of pertussis infection in young infants: a review of key evidence informing targeting of the cocoon strategy. Vaccine, 31(4), 618-625.
Wiley, K., Regan, A., & McIntyre, P. (2017). Immunisation and pregnancy–who, what, when and why?. Australian prescriber, 40(4), 122.
Wirsing von Koenig CH, Riffelmann M, Coenye T. Bordetella and related genera. In: Versalovic J, Carroll KC, Funke G, Jorgensen JH, Landry ML, Warnock, D.W., editors. Manual of Clinical Microbiology. 11th ed. Washington,D.C.: American Society for Microbiology; 2011.
World Health Organization. (2015). Pertussis vaccines: WHO position paper—August 2015. Weekly Epidemiological Record= Relevé épidémiologique hebdomadaire, 90(35), 433-458.
Yeung KHT, Duclos P, Nelson EAS, Hutubessy RCW. An update of the global burden of pertussis in children younger than 5 years: a modelling study. The Lancet Infectious Diseases. 2017;17(9):974-80.
**Reason for exclusion: no or no adequate comparison group (n=19)**
Datwani, H., Moro, P. L., Harrington, T., & Broder, K. R. (2015). Chorioamnionitis following vaccination in the vaccine adverse event reporting system. Vaccine, 33(27), 3110-3113.
Fortner, K. B., Edwards, K. M., Broder, K. R., Jimenez, N., Zhu, Y., Walter, E. B. & Swamy, G. K. (2016). 343: Reactogenicity of tetanus toxoid, reduced diphtheria toxoid, and acellular pertussis vaccine (Tdap) in pregnant women. American Journal of Obstetrics & Gynecology, 214(1), S193-S194.
Fortner, K. B., Swamy, G. K., Broder, K. R., Jimenez-Truque, N., Zhu, Y., Moro, P. L. & Yoder, S. (2018). Reactogenicity and immunogenicity of tetanus toxoid, reduced diphtheria toxoid, and acellular pertussis vaccine (Tdap) in pregnant and nonpregnant women. Vaccine, 36(42), 6354-6360.

Moro, P. L., Cragan, J., Lewis, P., & Sukumaran, L. (2017). Major birth defects after vaccination reported to the Vaccine Adverse Event Reporting System (VAERS), 1990 to 2014. Birth defects research, 109(13), 1057-1062.
Moro, P. L., Cragan, J., Tepper, N., Zheteyeva, Y., Museru, O., Lewis, P., & Broder, K. (2016). Enhanced surveillance of tetanus toxoid, reduced diphtheria toxoid, and acellular pertussis (Tdap) vaccines in pregnancy in the Vaccine Adverse Event Reporting System (VAERS), 2011–2015. Vaccine, 34(20), 2349-2353.
Perry, J., Towers, C. V., Weitz, B., & Wolfe, L. (2017). Patient reaction to Tdap vaccination in pregnancy. Vaccine, 35(23), 3064-3066.
Petousis-Harris, H., Walls, T., Watson, D., Paynter, J., Graham, P., & Turner, N. (2016). Safety of Tdap vaccine in pregnant women: an observational study. BMJ open, 6(4), e010911.
Regan AK, Tracey LE, Blyth CC, Richmond PC, Effler PV. A prospective cohort study assessing the reactogenicity of pertussis and influenza vaccines administered during pregnancy. Vaccine. 2016;34(20):2299-304.
Sukumaran, L., McCarthy, N. L., Kharbanda, E. O., Weintraub, E., Vazquez-Benitez, G., McNeil, M. M., ... & Lugg, M. M. (2015). Safety of tetanus, diphtheria, and acellular pertussis and influenza vaccinations in pregnancy. Obstetrics and gynecology, 126(5), 1069.
Talbot, E. A., Brown, K. H., Kirkland, K. B., Baughman, A. L., Halperin, S. A., & Broder, K. R. (2010). The safety of immunizing with tetanus–diphtheria–acellular pertussis vaccine (Tdap) less than 2 years following previous tetanus vaccination: experience during a mass vaccination campaign of healthcare personnel during a respiratory illness outbreak. Vaccine, 28(50), 8001-8007.
Vizzotti, C., Juarez, M. V., Bergel, E., Romanin, V., Califano, G., Sagradini, S.& Manzur, J. (2016). Impact of a maternal immunization program against pertussis in a developing country. Vaccine, 34(50), 6223-6228.
Vizzotti, C., Neyro, S., Katz, N., Juárez, M. V., Carrega, M. P., Aquino, A., & Fullone, F. K. (2015). Maternal immunization in Argentina: a storyline from the prospective of a middle income country. Vaccine, 33(47), 6413-6419.
Walls T, Graham P, Petousis-Harris H, Hill L, Austin N. Infant outcomes after exposure to Tdap vaccine in pregnancy: An observational study. BMJ Open 2016;6.
Walls, T., Graham, P., Petousis-Harris, H., Hill, L., & Austin, N. (2016). Infant outcomes after exposure to Tdap vaccine in pregnancy: an observational study. BMJ open, 6(1), e009536.
Wang, Min & Khromava, Alena & Mahmood, Asif & Dickson, Najla. (2011). Pregnant Women Receiving Tetanus-Diphtheria-Acellular Pertussis (Tdap) Vaccine: 6 Years of Adacel Vaccine Pregnancy Registry Data. Pharmacoepidemiology and Drug Safety. 20. S60-S61.
Wanlapakorn, N., Maertens, K., Chaithongwongwatthana, S., Srimuan, D., Suratannon, N., Vongpunsawad, S., ... & Poovorawan, Y. (2018). Assessing the reactogenicity of Tdap vaccine administered during pregnancy and antibodies to Bordetella pertussis antigens in maternal and cord sera of Thai women. Vaccine, 36(11), 1453-1459.
Winter K, Cherry JD, Harriman K. Effectiveness of prenatal versus postpartum tetanus, diphtheria, and acellular pertussis vaccination in preventing infant pertussis. Clin Infect Dis. 2017;64(1):3–8pmid:27624955
Winter, K., Zipprich, J., Harriman, K. Pertussis in California: A Tale of 2 Epidemics. Pediatr Infect Dis J Volume37, Number 4, April 2018
Zheteyeva, Y. A., Moro, P. L., Tepper, N. K., Rasmussen, S. A., Barash, F. E., Revzina, N. V. & Tokars, J. I. (2012). Adverse event reports after tetanus toxoid, reduced diphtheria toxoid, and acellular pertussis vaccines in pregnant women. American journal of obstetrics and gynecology, 207(1), 59-e1.
**Reason for exclusion: other interventions (n=13)**
Castagnini LA, Healy CM, Rench MA, Wootton SH, Munoz FM, Baker CJ. Impact of Maternal Postpartum Tetanus and Diphtheria Toxoids and Acellular Pertussis Immunization on Infant Pertussis Infection. Clin Infect Dis. 2012;54(1):78-84.
Leuridan, E., Hens, N., Peeters, N., de Witte, L., Van der Meeren, O., & Van Damme, P. (2011). Effect of a prepregnancy pertussis booster dose on maternal antibody titers in young infants. The Pediatric infectious disease journal, 30(7), 608-610.
Morgan, J. L., Baggari, S. R., McIntire, D. D., & Sheffield, J. S. (2015). Pregnancy outcomes after antepartum tetanus, diphtheria, and acellular pertussis vaccination. Obstetrics & Gynecology, 125(6), 1433-1438.
Munoz, F., & Englund, J. (2011). Infant pertussis: is cocooning the answer? Clin Infect Dis, 2011, 53:893 – 896
O'Connell, A., Tummon, A., Coleman, K., Jordan, A., McCormack, J., & Kelly, M. E. (2017). Antenatal Pertussis Vaccination: Why are General Practitioners Reluctant? A Mixed Methods StudySetting. Irish medical journal, 110(9), 634-634.
Quinn HE, Snelling TL, Habig A, Chiu C, Spokes PJ, McIntyre PB. Parental Tdap Boosters and Infant Pertussis: A Case-Control Study. Pediatrics. 2014;134(4):713-20.
Quinn, H. E., Snelling, T. L., Habig, A., Chiu, C., Spokes, P. J., & McIntyre, P. B. (2014). Parental Tdap boosters and infant pertussis: a case-control study. Pediatrics, 134(4), 713-720.

Shakib, J. H., Ralston, S., Raissy, H. H., Stoddard, G. J., Edwards, K. M., & Byington, C. L. (2010). Pertussis antibodies in postpartum women and their newborns. Journal of Perinatology, 30(2), 93.
Swamy, G. K., & Wheeler, S. M. (2014). Neonatal pertussis, cocooning and maternal immunization. Expert review of vaccines, 13(9), 1107-1114.
Winter K, Cherry JD, Harriman K. Effectiveness of prenatal versus postpartum tetanus, diphtheria, and acellular pertussis vaccination in preventing infant pertussis. Clin Infect Dis. 2017;64(1):3–8pmid:27624955
Wood, N., & Siegrist, C. A. (2011). Neonatal immunization: where do we stand? Current opinion in infectious diseases, 24(3), 190-195.
Wood, N., Marshall, H., White, O. J., Holt, P. G., & McIntyre, P. (2014). Antibody and cell-mediated immunity to pertussis 4 years after monovalent acellular pertussis vaccine at birth. The Pediatric infectious disease journal, 33(5), 511-517.
Wood, N., Nolan, T., Marshall, H., Richmond, P., Gibbs, E., Perrett, K., & McIntyre, P. (2018). Immunogenicity and safety of monovalent acellular pertussis vaccine at birth: a randomized clinical trial. *JAMA pediatrics*, *172*(11), 1045-1052.
**Reason for exclusion: conference publication (n=5)**
Bradley Layton J, Butler AM, Li D, Boggess KA, Becker-Dreps S. Safety of TDaP vaccination in pregnancy. Pharmacoepidemiology and Drug, Vol.25, 505-506
Dabrera G. Maternal pertussis vaccination is effective in preventing pertussis in infants: a case-control study in England and Wales, 2012-2013. ESCAIDE; 5-7 November, 2014; Stockholm: ECDC; 2014
Judy A., Singh A., Lee H., Gaskari S., Brodzinsky L., Vik J., et al. TDaP vaccination safety in pregnancy: A comparison of neonatal and obstetric outcomes among women receiving antepartum and postpartum vaccination. American Journal of Obstetrics and Gynecology. Conference: 35th Annual Meeting of the Society for Maternal-Fetal Medicine: The Pregnancy Meeting. San Diego, CA United States. Conference Publication: (var.pagings). 212 (1 SUPPL. 1) (pp S300-S301); Mosby Inc.; 2015.
Qiang Y, Chillarige Y, Said M, et al. Feasibility of using the Medicaid Statistical Information System (MSIS) to assess safety and effectiveness of 3rd trimester vaccination: A case study with tetanus toxoid, reduced diphtheria Toxoid and Acellular Pertussis (Tdap). Pharmacoepidemiology and Drug 2016;25:333.
Rega, A., Moore, H., De Klerk, N., & Effler, P. (2016, October). Maternal Vaccinations in Australia-Uptake, Safety and Impact. In AUSTRALIAN & NEW ZEALAND JOURNAL OF OBSTETRICS & GYNAECOLOGY (Vol. 56, pp. 13-14). 111 River St. Hoboken 07030-5774, NJ USA: Wiley-Blackwell.
**Reason for exclusion: commentary (n=4)**
Healy, C. M., Rench, M. A., & Baker, C. J. Reply to Leuridan et al. Clinical Infectious Diseases, Volume 57, Issue 3, 1 August 2013, Pages 472–473, https://doi.org/10.1093/cid/cit219
James D. Cherry, Editorial Commentary: The Effect of Tdap Vaccination of Pregnant Women on the Subsequent Antibody Responses of Their Infants, Clinical Infectious Diseases, Volume 61, Issue 11, 1 December 2015, Pages 1645–1647, https://doi.org/10.1093/cid/civ700
Millar, M. R., & Sanz, M. G. (2015). The administration of pertussis vaccine to pregnant women was associated with a small increased risk of chorioamnionitis, but not an increased risk of hypertensive disorders or preterm birth. BMJ Evidence-Based Medicine, 20(2), 73-73.
Sukumaran, L., & Omer, S. B. (2016). Repeat Tdap Vaccination and Adverse Birth Outcomes—Reply. *Jama*, *315*(12), 1286-1286.
**Reason for exclusion: case report or case series (n=3)**
Acosta, J., Benages, C., Díaz, M. A., Xiberta, M., & Muñiz, F. (2016). Preventing pertussis in the early infant: Development and results of a prenatal vaccination program. Acta Medica International, 3(2), 78.
Cabrera-Maqueda, J. M., Hernández-Clares, R., Baidez-Guerrero, A. E., Pío-Rendón, J. I. B., & Fernández, J. J. M. (2017). Optic neuritis in pregnancy after Tdap vaccination: Report of two cases. Clinical neurology and neurosurgery, 160, 116-118.
Maqueda, J. M. C., Ester, B. G. A., Clares, R. H., Luna, F. R., Ana, M. O., LALLANA, J. M., & Fernández, J. M. (2017). Optic neuritis during pregnancy following Tdpa vaccination: report of two cases. (P5. 188). Neurology, 88(16 Supplement), P5-188.

Table S2: Systematic review on safety and effectiveness of pertussis vaccination in pregnancy; results of the risk of bias (ROB) assessment in studies on safety and effectiveness outcomes

ROB for safety outcomes

**RCTs (Cochrane ROB tool)**	Sequence generation	Allocation concealment	Blinding	Selective outcome reporting	Incomplete outcome	Other sources of bias	Summary
Hoang et al., 2016 [1]	moderate	unclear	unclear	unclear	unclear	high	high
Munoz et al., 2014 [2]	low	low	low	low	low	low	low
Halperin et al., 2018 [3]	low	low	low	low	low	moderate	moderate

**Observational studies (Robins-I tool)**	Bias due to confounding	Bias in selection of participants into the study/analysis	Bias in classification of interventions	Bias due to deviations from intended interventions	Bias due to missing data	Bias in measurement of outcomes	Bias in selection of reported result	Summary
Berenson et al., 2016 [4]	serious	serious	serious	serious	serious	serious	moderate	serious
DeSilva et al., 2016 [5]	serious	serious	serious	moderate	serious	moderate	moderate	serious
DeSilva et al., 2017 [6]	serious	serious	serious	low	serious	serious	moderate	serious
Donegan et al., 2014 [7]	critical	serious/ critical	moderate/ serious	moderate/ serious	serious	moderate	moderate	critical
Griffin et al., 2018 [8]	serious	moderate	serious	low	moderate	serious	low	serious
Kharbanda et al., 2014 [9]	serious	serious	serious	low	serious	serious	moderate	serious
Kharbanda et al., 2016 [10]	critical	critical	moderate	low	serious	moderate	serious	critical
Layton et al., 2017 [11]	serious	serious	moderate	serious	low	serious	moderate	serious
Maertens et al., 2016 [12]	critical	serious	serious	serious	serious	serious	low	critical
Morgan et al., 2015 [13]	serious	moderate	moderate	low	low	moderate	mod./ serious	serious
Shakib et al., 2013 [14]	serious	serious	unclear	low	low	moderate	moderate	serious

ROB for effectiveness outcomes

**Observational studies (Robins-I tool)**	Bias due to confounding	Bias in selection of participants into the study/analysis	Bias in classification of interventions	Bias due to deviations from intended interventions	Bias due to missing data	Bias in measurement of outcomes	Bias in selection of reported result	Summary
Amirthalingam et al., 2014 [15]	serious	moderate	low	low	low	low	low	serious
Amirthalingam et al., 2016 [16]	serious	unclear	low	low	low	low	low	serious
Baxter et al., 2017 [17]	moderate	unclear	low	low	unclear	low	low	moderate
Becker-Dreps et al., 2018 [18]	serious	moderate	moderate	low	low	moderate/serious*	moderate	serious
Bellido-Blasco et al., 2017 [19]	moderate	moderate	low	low	low	low	low	moderate
Dabrera et al., 2018 [20]	serious	moderate	low	low	unclear	low	low	serious
Saul et al., 2017 [21]	moderate	moderate	low	low	moderate	low	low	moderate
Skoff et al., 2017 [22]	moderate	serious	low	low	moderate	low	low	serious

*serious for "pertussis", moderate for "inpatient pertussis"

Table S3: Systematic review on safety and effectiveness of pertussis vaccination in pregnancy; GRADE Evidence profile

Quality assessment							No of patients		Effect		Quality	Importance
**No of studies**	**Design**	**Risk of bias (ROBINS-I for NRS)**	**Inconsistency**	**Indirectness**	**Imprecision**	**Other considerations**	**Pertussis vaccination during pregnancy**	**No vaccination**	**Relative (95% CI)**	**Absolute**		
**VE: Pertussis in infants <3 months (cohort studies) (assessed with: lab-confirmed pertussis)**												
1 (Amirthalingam et al., 2016 [16])	observational study	serious[1]	no serious inconsistency	no serious indirectness	no serious imprecision	none	35/243 (14.4%)	49327/72538 (68%)[2]	VE 91 (88 to 96)	0 more per 1000 (from 1000 more to 1000 more)[3]	⊕⊕OO LOW	CRITICAL
**VE: Pertussis in infants <3 months (case-control studies) (assessed with: lab-confirmed pertussis)**												
2 (Bellido-Blasco et al. [19], 2017 [19]; Saul et al., 2017 [21])	observational studies	moderate[4]	serious[5]	no serious indirectness	no serious imprecision	none	70 cases 114 controls		VE ranged from 69 to 91	-	⊕⊕OO LOW	CRITICAL
								0%		-		
**VE: Hospitalization (timing of exposure 3 months; assessed with: lab-confirmed pertussis)**												
1[6] (Saul et al., 2017 [21])	observational study	moderate	no serious inconsistency	no serious indirectness	no serious imprecision	none	37 cases 37 controls		VE 94 (59 to 99)	-	⊕⊕⊕O MODERATE	CRITICAL
								0%		-		
**VE: Pertussis related death during first year of life (assessed with: lab. confirmed pertussis)**												
1 (Amirthalingam et al., 2016 [16])	observational study	serious[1]	no serious inconsistency	no serious indirectness	no serious imprecision	none	1/11 (9.1%)	158/232 (68.1%)	VE 95 (79 to 100)	1000 more per 1000 (from 1000 more to 1000 more)	⊕⊕OO LOW	CRITICAL

**Fever >38°C after vaccination (assessed with: medically attended fever in 3 days post vaccination)**												
1[7] (Kharbanda et al., 2016 [10])	observational studies	serious[8]	no serious inconsistency	no serious indirectness	no serious imprecision	none	15/53885 (0.03%)	6/109253 (0.005%)	RR 2.16 (1.7 to 2.8)[9]	64 more per 1,000,000 (from 38 more to 99 more)	⊕⊕OO LOW	IMPORTANT
							alternative scenarios:	double the observed risk: 0.010%		116 more per 1,000,000 (from 70 more to 180 more)		
								Four times the observed risk: 0.02%		232 more per 1,000,000 (from 140 more to 360 more)		
**Preterm birth (assessed with: <37 weeks of gestation)**												
3[10] (Kharbanda et al., 2014 [9]; Berenson et al., 2016 [4]; Griffin et al., 2018 [8])	observational studies	serious[11]	no serious inconsistency	no serious indirectness	serious[12]	none	-	0%	RR ranged from 0.68 to 1.03	-	⊕OOO VERY LOW	CRITICAL
**Stillbirth**												
4 (Berenson et al., 2016 [4]; Donegan et al., 2014 [7]; Morgan et al. [13], 2015 ; Shakib et al., 2013 [14])	observational studies	serious[13]	no serious inconsistency	no serious indirectness	no serious imprecision	none	-	-	RR ranged from 0.36 to 0.85	-	⊕⊕OO LOW	CRITICAL
								0%		-		
**Pre-eclampsia (assessed with varying definitions)**												
2[14] (Kharbanda et al., 2014 [9]; Layton et al., 2017 [11])	observational studies	serious	no serious inconsistency	no serious indirectness	no serious imprecision	none	-	-	RR ranged from 0.96 to 1.09	-	⊕⊕OO LOW	CRITICAL
								0%		-		
**Low birth weight (assessed with: <2500g)**												
1[15] (Berenson et al., 2016	observational	serious	no serious	no serious	no serious	none	61/1109	59/650	RR 0.76 (0.51 to	22 fewer per 1000 (from 44	⊕⊕OO	CRITICAL

[4])	study		inconsistency	indirectness	imprecision		(5.5%)	(9.1%)	1.14)	fewer to 13 more)	LOW	
							alternative scenarios:	5%		12 fewer per 1000 (from 25 fewer to 7 more)		
								15%		36 fewer per 1000 (from 74 fewer to 21 more)		
**Chorioamnionitis (assessed with: ICD9, ICD10, others)**												
6[16] (Kharbanda et al., 2014 [9]; Berenson et al., 2016 [4]; Layton et al., 2017 [11] ; Griffin et al., 2018 [8]; DeSilva et al., 2017 [6] ; Morgan et al., 2015 [13])	observational studies	serious[13]	no serious inconsistency	no serious indirectness	no serious imprecision	none	637/11351 (5.6%)	5329/97265 (5.5%)	RR ranged from 1.1 to 1.53	-	⊕⊕OO LOW	IMPORTANT
**Malformations (assessed with: varying definitions)**												
2[15,17] Berenson et al., 2016 [4]; DeSilva et al., 2016 [5])	observational studies	serious[13]	no serious inconsistency	no serious indirectness	no serious imprecision	none	-	-	RR ranged from 0.8 to 0.98	-	⊕⊕OO LOW	CRITICAL
								0%		-		
**NIC (assessed with: NICU admission in 30 days post-delivery or not defined)**												
1 (Layton et al., 2017 [11])	observational study	serious[13]	no serious inconsistency	no serious indirectness	no serious imprecision	none	6996/80217 (8.7%)	42904/543906 (7.9%)	RR 1.0 (0.97 to 1.03)	0 fewer per 1000 (from 2 fewer to 2 more)	⊕⊕OO LOW	CRITICAL
							alternative scenarios:	3%		0 fewer per 1000 (from 1 fewer to 1 more)		
								10%		0 fewer per 1000 (from 3 fewer to 3 more)		

**Neonatal death (assessed with: Neonatal death within 7 days of delivery or not defined)**												
2 (Morgan et al., 2015 [13]; Donegan et al., 2014 [7])	observational studies	very serious[18]	serious[19]	no serious indirectness	no serious imprecision	none	-	-	RR ranged from 0.16 to 1	-	⊕OOO VERY LOW	CRITICAL
								0%		-		
**Sepsis**												
0	No evidence available					none	-	-	-	-		IMPORTANT
								0%		-		

[1] Use of screening method for calculation of VE; serious ROB
[2] Crude estimate only, because screening method was used.
[3] Cannot be calculated since screening method was used
[4] Both rated as moderate RoB.
[5] Wide range of effect estimates in the studies
[6] Case-control study with lowest risk of bias (Saul et al.) was used here. One additional case-control study (Skoff et al.) and one cohort study (Becker-Dreps et al.) had higher risk of bias.
[7] Largest cohort study with control group for this outcome (based on VSD data; Kharbanda, 2016)
[8] RoB was rated serious because only an unadjusted estimate was reported.
[9] Crude ratio effect estimate (95% CI); authors do not present adjusted results, only for composite outcome
[10] Three cohort studies (Kharbanda et al., Berenson et al., Griffith et al.) with confounder-adjusted estimates.
[11] All 3 were rated serious risk of bias.
[12] Adjusted effects varied and partly included 1.0
[13] Serious RoB in all studies due to various categories
[14] Three cohort studies reported confounder-adjusted estimates.
[15] One study with plausible background rate and confounder-adjusted estimate.
[16] 5 cohort studies reported confounder-adjusted estimates.
[17] Two studies with confounder-adjusted estimates.
[18] Only crude estimates reported.
[19] very low case numbers, very wide 95%Cis

References

1. Hoang HTT, Leuridan E, Maertens K, Nguyen TD, Hens N, Vu NH, Caboré RN, Duong HT, Huygen K, Van Damme P *et al*: **Pertussis vaccination during pregnancy in Vietnam: Results of a randomized controlled trial Pertussis vaccination during pregnancy**. *Vaccine* 2016, **34**(1):151-159.
2. Munoz FM, Bond NH, Maccato M, Pinell P, Hammill HA, Swamy GK, Walter EB, Jackson LA, Englund JA, Edwards MS *et al*: **Safety and Immunogenicity of Tetanus Diphtheria and Acellular Pertussis (Tdap) Immunization During Pregnancy in Mothers and Infants: A Randomized Clinical Trial**. *JAMA* 2014, **311**(17):1760-1769.
3. Halperin S, Langley J, Ye L, MacKinnon-Cameron D, Elsherif M, Allen V, Smith B, Halperin B, McNeil S, Vanderkooi O *et al*: **A Randomized Controlled Trial of the Safety and Immunogenicity of Tetanus, Diphtheria, and Acellular Pertussis Vaccine Immunization During Pregnancy and Subsequent Infant Immune Response.** *Clin Infect Dis 2018 Sep 14;67(7):1063-1071* 2018.
4. Berenson AB, Hirth JM, Rahman M, Laz TH, Rupp RE, Sarpong KO: **Maternal and infant outcomes among women vaccinated against pertussis during pregnancy**. *Human and Immunotherapeutics* 2016, **12**(8):1965-1971.
5. DeSilva M, Vazquez-Benitez G, Nordin JD, Lipkind HS, Romitti PA, DeStefano F, Kharbanda EO: **Tdap vaccination during pregnancy and microcephaly and other structural birth defects in offspring**. *JAMA - Journal of the American Medical Association* 2016, **316**(17):1823-1825.
6. DeSilva M, Vazquez-Benitez G, Nordin JD, Lipkind HS, Klein NP, Cheetham TC, Naleway AL, Hambidge SJ, Lee GM, Jackson ML *et al*: **Maternal Tdap vaccination and risk of infant morbidity**. *Vaccine* 2017, **35**(29):3655-3660.
7. Donegan KKBBP: **Safety of pertussis vaccination in pregnant women in UK: observational study**. *BMJ* 2014, **349**.
8. Griffin JB, Yu L, Watson D, Turner N, Walls T, Howe AS, Jiang Y, Petousis-Harris H: **Pertussis Immunisation in Pregnancy Safety (PIPS) Study: A retrospective cohort study of safety outcomes in pregnant women vaccinated with Tdap vaccine**. *Vaccine* 2018, **36**(34):5173-5179.
9. Kharbanda EO, Vazquez-Benitez G, Lipkind HS, et al.: **Evaluation of the association of maternal pertussis vaccination with obstetric events and birth outcomes**. *JAMA* 2014, **312**(18):1897-1904.
10. Kharbanda EO, Vazquez-Benitez G, Lipkind HS, Klein NP, Cheetham TC, Naleway AL, Lee GM, Hambidge S, Jackson ML, Omer SB *et al*: **Maternal Tdap vaccination: Coverage and acute safety outcomes in the vaccine safety datalink, 2007–2013**. *Vaccine* 2016, **34**(7):968-973.
11. Layton JB, Butler AM, Li D, Boggess KA, Weber DJ, McGrath LJ, Becker-Dreps S: **Prenatal Tdap immunization and risk of maternal and newborn adverse events**. *Vaccine* 2017, **35**(33):4072-4078.
12. Maertens K, Caboré RN, Huygen K, Hens N, Van Damme P, Leuridan E: **Pertussis vaccination during pregnancy in Belgium: Results of a prospective controlled cohort study**. 2016, **34**(1):142-150.
13. Morgan JL, Baggari SR, McIntire DD, Sheffield JS: **Pregnancy Outcomes After Antepartum Tetanus, Diphtheria, and Acellular Pertussis Vaccination**. *Obstet Gynecol* 2015, **125**(6).
14. Shakib JH, Korgenski K, Sheng X, Varner MW, Pavia AT, Byington CL: **Tetanus, Diphtheria, Acellular Pertussis Vaccine during Pregnancy: Pregnancy and Infant Health Outcomes**. *The Journal of Pediatrics* 2013, **163**(5):1422-1426.e1424.

15. Amirthalingam G, Andrews N, Campbell H, Ribeiro S, Kara E, Donegan K, Fry NK, Miller E, Ramsay M: **Effectiveness of maternal pertussis vaccination in England: an observational study**. *The Lancet* 2014, **384**(9953):1521-1528.
16. Amirthalingam G, Campbell H, Ribeiro S, Fry NK, Ramsay M, Miller E, Andrews N: **Sustained effectiveness of the maternal pertussis immunization program in England 3 years following introduction**. *Clin Infect Dis* 2016, **63**:S236-S243.
17. Baxter R, Bartlett J, Fireman B, Lewis E, Klein NP: **Effectiveness of vaccination during pregnancy to prevent infant pertussis**. *Pediatrics* 2017, **139**(5).
18. Becker-Dreps S, Butler AM, McGrath LJ, Boggess KA, Weber DJ, Li D, Hudgens MG, Layton JB: **Effectiveness of Prenatal Tetanus, Diphtheria, Acellular Pertussis Vaccination in the Prevention of Infant Pertussis in the U.S**. *Am J Prev Med* 2018, **55**(2):159-166.
19. Bellido-Blasco J, Guiral-Rodrigo S, Miguez-Santiyan A, Salazar-Cifre A, Gonzalez-Moran F: **A case-control study to assess the effectiveness of pertussis vaccination during pregnancy on newborns, Valencian community, Spain, 1 March 2015 to 29 February 2016**. *Euro surveillance : bulletin Europeen sur les maladies transmissibles = European communicable disease bulletin* 2017, **22**(22).
20. Dabrera G, Amirthalingam G, Andrews N, Campbell H, Ribeiro S, Kara E, Fry NK, Ramsay M: **A case-control study to estimate the effectiveness of maternal pertussis vaccination in protecting newborn infants in England and Wales, 2012-2013**. *Clin Infect Dis* 2014, **60**(3):333-337.
21. Saul N, Wang K, Bag S, Baldwin H, Alexander K, Chandra M, Thomas J, Quinn H, Sheppeard V, Conaty S: **Effectiveness of maternal pertussis vaccination in preventing infection and disease in infants: The NSW Public Health Network case-control study**. *Vaccine* 2018.
22. Skoff TH, Blain AE, Watt J, Scherzinger K, McMahon M, Zansky SM, Kudish K, Cieslak PR, Lewis M, Shang N *et al*: **Impact of the US Maternal Tetanus, Diphtheria, and Acellular Pertussis Vaccination Program on Preventing Pertussis in Infants <2 Months of Age: A Case-Control Evaluation**. *Clin Infect Dis* 2017, **65**(12):1977-1983.
